# 2-Furoic acid associated with the infection of nematodes by *Dactylellina haptotyla* and its biocontrol potential on plant root-knot nematodes

**DOI:** 10.1128/spectrum.01896-23

**Published:** 2023-09-27

**Authors:** Hong-Mei Lei, Jun-Tao Wang, Qian-Yi Hu, Chun-Qiang Li, Ming-He Mo, Ke-Qin Zhang, Guo-Hong Li, Pei-Ji Zhao

**Affiliations:** 1 State key Laboratory for Conservation and Utilization of Bio-Resources in Yunnan, School of Life Sciences, Yunnan University, Kunming, Yunnan, China; Chinese Academy of Sciences, Shanghai, China

**Keywords:** nematode-trapping fungi, *Dactylellina haptotyla*, infection, genomics, metabolomic, 2-furoic acid

## Abstract

*Dactylellina haptotyla* is a typical nematode-trapping fungus that has garnered the attention of many scholars for its highly effective lethal potential for nematodes. Secondary metabolites play an important role in *D. haptotyla*-nematode interactions, but which metabolites perform which function remains unclear. We report the metabolic functions based on high-quality, chromosome-level genome assembly of wild *D. haptotyla* YMF1.03409. The results indicate that a large variety of secondary metabolites and their biosynthetic genes were significantly upregulated during the nematode-trapping stage. In parallel, we identified that 2-furoic acid was specifically produced during nematode trapping by *D. haptotyla* YMF1.03409 and isolated it from fermentation production. 2-Furoic acid demonstrated strong nematicidal activity with an LD_50_ value of 55.05 µg/mL against *Meloidogyne incognita* at 48 h. Furthermore, the pot experiment showed that the number of galls of tomato root was significantly reduced in the experimental group treated with 2-furoic acid. The considerable increase in the 2-furoic acid content during the infection process and its virulent nematicidal activity revealed an essential synergistic effect during the process of nematode-trapping fungal infection.

**IMPORTANCE:**

*Dactylellina haptotyla* have significant application potential in nematode biocontrol. In this study, we determined the chromosome-level genome sequence of *D. haptotyla* YMF1.03409 by long-read sequencing technology. Comparative genomic analysis identified a series of pathogenesis-related genes and revealed significant gene family contraction events during the evolution of *D. haptotyla* YMF1.03409. Combining transcriptomic and metabolomic data as well as *in vitro* activity test results, a compound with important application potential in nematode biocontrol, 2-furoic acid, was identified. Our result expanded the genetic resource of *D. haptotyla* and identified a previously unreported nematicidal small molecule, which provides new options for the development of plant biocontrol agents.

## INTRODUCTION

Plant-parasitic nematodes are major agricultural pests that cause devastation in agriculture around the world. The annual global economic losses caused by plant-parasitic nematodes are estimated to be between $80 and $173 billion (1, 2). Nematode-trapping fungi are natural nematode predators with enormous application potential in managing plant-parasitic nematode populations and lowering agricultural losses (3, 4). Nematode-trapping fungi mainly live saprophytically in soil. The formation of a trap is a signature event of nematode-trapping fungi from the saprophytic to parasitic stages (5, 6); it can be produced spontaneously or induced in the presence of nematodes or some metabolites (7). As the most essential weapon for nematode-trapping fungi, the structure of the trap is diverse (8), resulting in substantial variances in its predation process. Nematode predation by nematode-trapping fungi is a complex and delicate process (7, 9). Despite the fact that several research have been conducted on this predating process, many crucial questions remain unanswered.

Microbial metabolites are mediators of biological communication and competition between microorganisms and the environment (10, 11). These metabolites, formed by various enzymatic mechanisms that have evolved over thousands of years, contribute to biomass production and environmental niche assurance (12, 13). During interactions between pathogens (or parasites) and hosts, metabolites produced by the pathogens (or parasites) often play key roles in the infestation process (14, 15). In 1995, Anderson et al. reported three new derivatives of oligosporon from nematode-trapping fungus *Orbilia oligospora* (*Arthrobotrys oligospora*), which showed toxic activity against intestinal parasitic nematodes (16). Researchers have also obtained a series of oligosporons and arthrosporols from *O. oligospora* that possess the ability to modulate the differentiation of traps (17 – 19). In 2013, Wu et al. isolated paganins A and B from *O. oligospora* and found that the compounds could promote trap generation (18). A volatile compound, methyl 3-methyl-2-butenoate, obtained from *O. oligospora*, can attract a variety of nematodes, including *Caenorhabditis elegans* (20). Additionally, *Arthrobotrys flagrans* (*Duddingtonia flagrans*) produce a polyketone 6-methyl salicylic acid that regulates the spatiotemporal control of trap formation and acts as a chemoattractant to *C. elegans* (21). The discovery of these metabolites and the resolution of their activity have improved our understanding of nematode-trapping fungi. In the 1960s, Olthof and Estey tested the activity of filtrates of *O. oligospora* that had already infected nematodes and found that many worms became immobile and appeared dead (22). Therefore, they speculated that nematode-trapping fungi can generate metabolites that play a synergistic role in the infection process.

**Fig 1:**
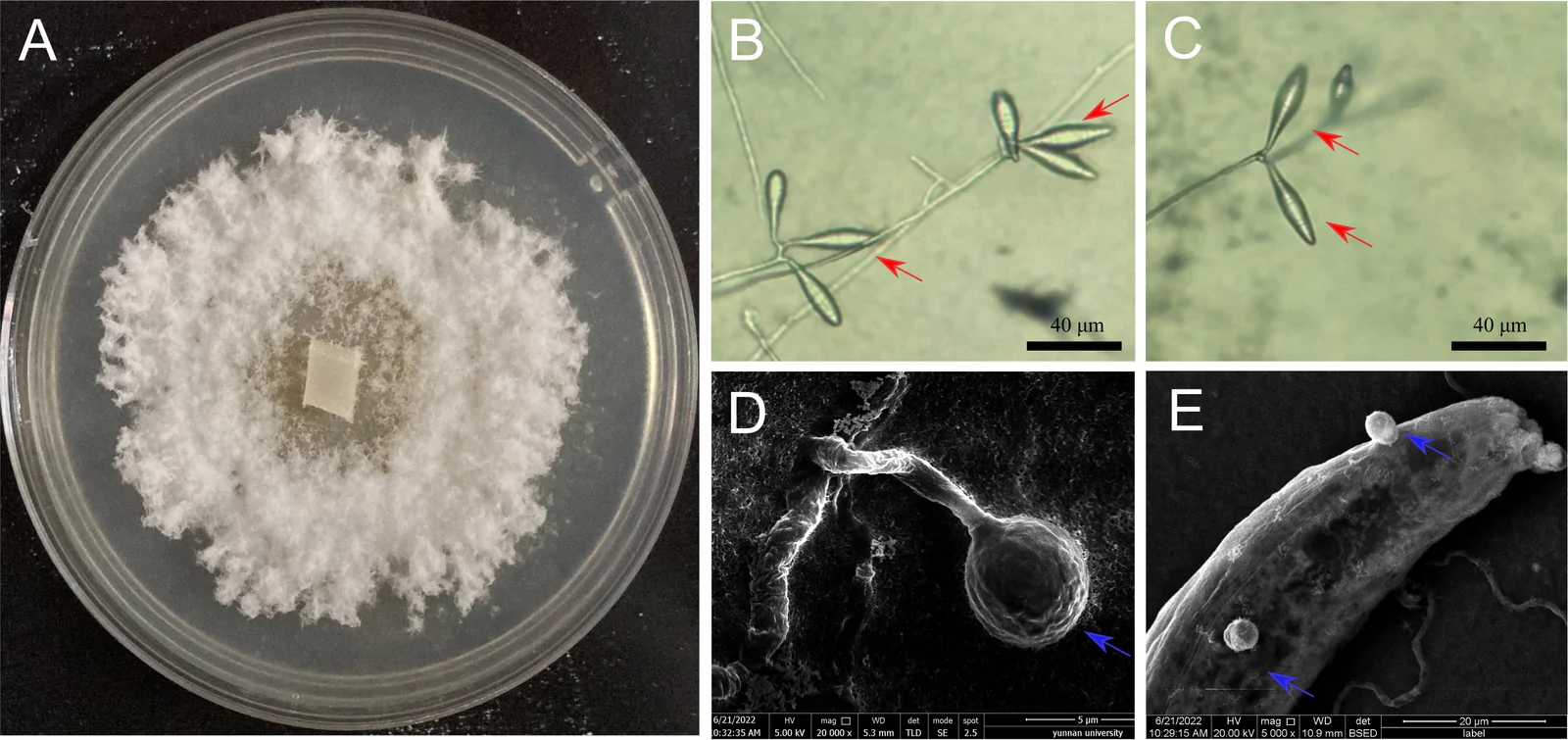
The characterizations of *D. haptotyla* YMF1.03409. (**A**) The growth status of *D. haptotyla* YMF1.03409 on PDA. (**B**) and (**C**) The conidiophores of *D. haptotyla* YMF1.03409, which are marked by red arrows. (**D**) and (**E**) The knobs of *D. haptotyla* YMF1.03409, which are marked by blue arrows.

*Dactylellina haptotyla* is a representative species of nematode-trapping fungi. Its spores are poke-shaped; it can capture its prey and enter the parasitic stage by forming knobs (Fig. 1). Comparative genomic analysis suggests that knob formation is an evolutionary tendency of morphological simplicity and efficiency enhancement (23, 24). The high pathogenicity of *D. haptotyla* suggests that it may have huge application potential in the biological control of nematodes (25, 26). In a previous study of *D. haptotyla*, Tunlid and his group provided several excellent results: after studying differentially expressed genes (DEGs) between traps and hyphae, as well as transcriptome and proteome data during *D. haptotyla* infection, they identified several extended-domain highly expressed families, including subtilisin, common in several fungal extracellular membrane proteins, the DUF3129 family (*gas1*), small secreted proteins, and cell surface adhesins. Most of them are known to be associated with the pathogenicity of pathogenic fungi (5, 26, 27). The genomic mechanism of parasitic evolution of predatory nematode fungi was elucidated through comparative genomics and transcriptomics. These findings provide a wealth of data for further understanding the function of *D. haptotyla* infection-associated metabolites (28).

Biosynthetic gene clusters (BGCs) can be predicted from microbial genome analysis (e.g., antiSMASH and PRISM) through the reporting of large-scale genomes and the outcomes of big data in natural product research. The quality of assembly and annotation has a considerable influence on the outcomes of genome-based research since assembled and annotated genome sequences frequently serve as the raw material for genome mining (29). The integrity of gene content and continuity within the chosen genome sequence heavily influence our ability to comprehend the diversity of BGCs (30). Long-read technology can obtain a more complete and continuous genome assembly (31). In this study, we obtained high-quality genome assembly at the chromosome level of *D. haptotyla* YMF1.03409 by high-throughput sequencing using Hi-C technology and constructed the first metabolome landscape of this fungus. Furthermore, we discovered some unique antinematode molecular features of *D. haptotyla* YMF 1.03409, such as *EVM08G003990.1*, which is located in the shikimic acid pathway, and its expression level was significantly increased during reciprocal interaction. We also discovered that the small molecule compounds (such as 2-furoic acid) were also specifically present in the reciprocal metabolome samples; nematicidal activity experiments demonstrated the tremendous nematode lethality of 2-furoic acid. Additionally, by comparative genomic analysis, we found that *D. haptotyla* YMF1.03409 exhibited a significant gene family contraction, which may be because *D. haptotyla* YMF1.03409 discarded several genes not associated with parasitism during evolution.

## MATERIALS AND METHODS

### Fungal, nematode materials, and growth conditions

The strain used in this study was *D. haptotyla* YMF1.03409, which was maintained at the State Key Laboratory for Conservation and Utilization of Bio-Resources and Key Laboratory for Microbial Resources. *D. haptotyla* YMF1.03409 was routinely cultured on potato dextrose agar (PDA) plates. Mycelium samples for genome sequencing were provided in the supplemental material. The nematodes used in this study were *Panagrellus redivivus*, *Meloidogyne incognita,* and *C. elegans. P. redivivus* was routinely cultured in an oat medium at 25°C. *M. incognita* was obtained by picking nematode egg masses from the roots of infested plants that were then incubated at 25°C. *C. elegans* was routinely cultured in a nematode growth medium at 25°C, and *Escherichia coli* OP50 was used as the food source.

The methods of genome sequencing, assembly, gene and genomic component prediction, and comparative genomic analysis are provided in the supplemental material.

### RNA-seq and analysis

The strains were cultured on PDA plates at 28°C for 10 days. We then collected the spores and incubated them in 1% sucrose plates at 28°C for 3 days until spore germination. For the treated group, approximately 150 nematodes (*P. redivivus*) were added to the plates, and we sampled two time points (24 and 48 h after treatment), labeled as PD24 and PD48. *D. haptotyla* YMF1.03409 and *P. redivivus* were grown in parallel under identical conditions and used as control groups, labeled as D24, D48, P24, and P48, respectively. The transcriptome analysis time point was chosen based on the infection status of *D. haptotyla* YMF1.03409. The beginning, middle, and end of the infestation were determined to be 12, 24, and 48 h following the addition of nematodes, respectively. The nematode infectious time was also adjusted as a covariate. The nematode mortality rate and quantity of adhesive knobs considerably increased over the course of the infection. Nearly all nematodes perished by the conclusion of the infestation (48 h after nematode inoculation), and knobs counts peaked (Fig. S1 and S2).

The samples were collected, and RNA was extracted using the TRIzol method. The RNA-seq analysis method is described in the supplemental material.

### Metabolomic sample preparation from the infestation of *P. redivivus* by *D. haptotyla* YMF1.03409

Aliquots (600 µL) of conidial suspension were spread on 6 cm Petri dishes with water agar (WA) medium and incubated at 28°C for 3 days until conidia germination. Approximately 150 nematodes (*P. redivivus*) were added to the middle of the WA plates, and the fungus-nematode interaction samples were collected at 24 and 48 h (after the injection of *P. redivivus*) and marked as PD24 and PD48, respectively. The nematodes and fungi used for the control were prepared in the same way as described above and marked as P24, P48, D24, and D48, respectively. The interaction, nematode, and fungus samples were harvested separately and extracted with 200 mL of organic reagent mixture (ethyl acetate:methanol:acetic acid = 80:15:5, vol/vol/vol) for 3 days. The extract liquids were separated by filtration, and the filtrate was concentrated by drying under vacuum. Each dried sample was resuspended in 2 mL of chromatographic methanol, filtered through 0.22 µm Steritop units (Millipore), and stored at 4°C prior to liquid chromatography-mass spectrometry (LC-MS) analyses. Metabolomic data acquisition and statistical analysis are presented in the supplemental material.

### Purification and characterization of 2-furoic acid from *D. haptotyla* YMF1.03409

After fermentation on rice medium [60 g of rice, 0.3 g of (NH_4_)_2_SO_4_ and pork liver, and 30 mL of H_2_O]. After separation and purification using various chromatography techniques, 14 compounds were ultimately isolated after the crude extract (35.64 g) was obtained using an organic solvent. The fermentation, extraction, isolation, and spectroscopic data are presented in the supplemental material.

### Virulence assay

To understand the virulence of 2-furoic acid, we conducted a nematicidal activity test. In particular, a certain concentration of 2-furoic acid solution was prepared and added to 3.5 cm plates, 2 mL per plate. Subsequently, 100–150 nematodes (*M. incognita*, *P. redivivus*, and *C. elegans*) were added to the plates. The number of dead and live nematodes was recorded after 12, 24, and 48 h under a light microscope (Olympus). The assay was conducted in triplicate (an aqueous solution under the same conditions served as the control group).

### Root-knot nematode infestation experiment

Three treatment groups were used in this experiment: (i) positive control avermectin solution, (ii) negative control aqueous solution, and (iii) experimental group 2-furoic acid solution. The soil weighed approximately 80 g, with three tomato seedlings per pot. Before transplanting the tomato seedlings, the roots (substrate soil containing the roots) were soaked in each of the three groups of solutions at a concentration of 350 µg/mL for 10 min and then transplanted into pots. Three hundred milliliters of the corresponding treatment group were irrigated to the tomato roots of each experimental pot after transplanting and watered normally at a later stage. After two days, *M. incognita* was added, and the number of *M. incognita* was approximately 5,000 per pot. Normal watering was conducted during the later period. Tomato plants from each treatment group were removed after 20 and 35 days, and the roots were observed and counted for root-knot nematode parasitism after the soil was gently rinsed off from the roots.

## RESULTS

### Chromosome level genome of *D. haptotyla* YMF 1.03409

**Fig 2:**
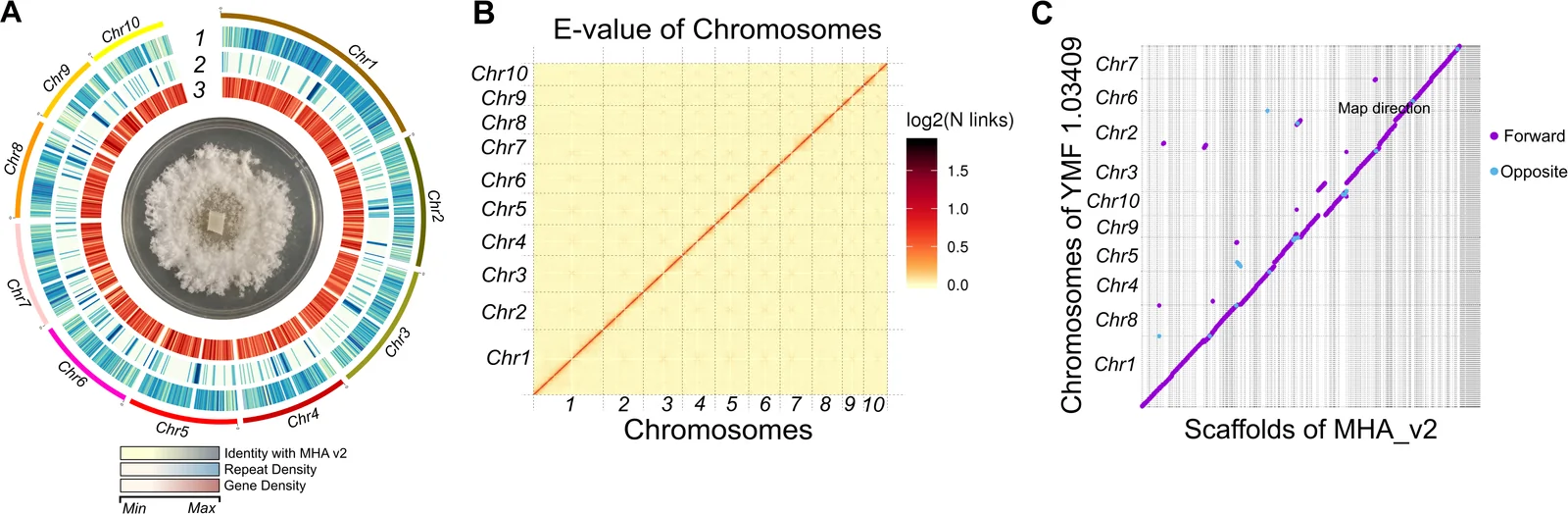
The new chromosome genome assembly of *D. haptotyla* YMF1.03409. (**A**) Circos plot of *D. haptotyla* YMF1.03409. The outer to inner circles represent the mapping identity with MHA_v2, repetitive region density, and gene density, respectively. (**B**) The Hi-C heatmap of *D. haptotyla* YMF1.03409 indicates the satisfactory quality of the assembly. (**C**) The dotplot for genome-wide alignment between MHA_v2 and *D. haptotyla* YMF1.03409.

We extracted DNA from the *D. haptotyla* YMF1.03409 strain, which was isolated from China, for sequencing and genome assembly. A total of 6.99 Gbp clean sequencing data were obtained using nanopore sequencing technology from Oxford Nanopore Technologies. Nanopore long reads were then assembled into 11 contigs with an N50 length of 3,741,584 bp. We then conducted Illumina short-read sequencing with an average depth of approximately 123× to polish the contigs generated by long-read sequencing. The short-read remapping ratio was 98.68% and covered 99.7% of the contigs, which demonstrated that our primary assembly was of high quality. The contigs were then anchored to the chromosome model using Hi-C data (7.41 Gbp clean data; Fig. 2B). The mapping rate was 93.58%, and 80.16% of the Hi-C reads were uniquely mapped to the primary contigs. The mapped reads were used to cluster, orientate, and order the contigs to the chromosomes. Finally, we successfully constructed a chromosome-level assembly of *D. haptotyla* YMF1.03409 with a size of 39.55 Mbp (Fig. 2A; Table S1, GenBank assembly accession: JAQJAX000000000).

The assembly of *D. haptotyla* YMF1.03409 showed higher sequence identity with the previously published *D. haptotyla* CBS 200.50 genome assembly MHA_v2 (GenBank assembly accession: GCA_000441935.1) (28), which was constructed by 454 pyrosequencing method (Fig. 2C). The one-to-one comparative analysis indicated an improvement in the chromosome-level assembly (Table 1). The *D. haptotyla* YMF1.03409 assembly has a longer total length (39.55 Mb vs 39.53 Mb) and a higher level of completeness (98.0% vs 97.6%). The identified genes were more than those in the previous *D. haptotyla* CBS 200.50 genome assembly MHA_v2 (11,073 vs 10,959). The number of transposable elements identified was also higher than that in the previous version (408 vs 314, Table S2).

**TABLE 1:** Statistics of *D. haptotyla* CBS 200.50 assembly and *D. haptotyla* YMF1.03409 assembly

	*D. haptotyla* CBS 200.50	*D. haptotyla* YMF1.03409
Sequencing platform	454 pyrosequencing technology	Nanopore + NGS + Hi-C
Assembly size (bp)	39,531,920	39,557,028
Depth	28×	176.60 (SMRT^*a*^) + 123.21× (NGS^*b*^)
GC content (%)	45.30	45.44
Protein-coding genes	10,959	11,073
Number of scaffolds	1,279	10
BUSCO completeness (%)	97.6	98.0
Longest sequence length (bp)	937,461	7,783,607

^*a*^ SMRT, single molecule real-time sequencing.

^*b*^ NGS, next-generation sequencing.

### Pathogenic genes widely existed in *D. haptotyla* YMF1.03409 genome

To provide a general view of potentially pathogenic genes in the nematode-trapping fungi, the basic local alignment search tool was used on the entire *D. haptotyla* YMF1.03409 genome against the pathogen-host interaction database (PHI-base). Among the 2,727 genes matching a PHI-base entry, 47% of the mutant phenotype of those genes was annotated as “reduced virulence” or “loss of pathogenicity.” A large number of virulence genes showed the capacity of *D. haptotyla* YMF1.03409 to control nematodes (Table S3).

The *D. haptotyla* YMF1.03409 genome contains abundant genes related to the fungal lifestyle. Carbohydrate-active enzymes (CAZYs) are involved in nutrient sensing and acquisition and in determining the saprophytic ability of fungi (32). Information about CAZYs could provide insights into the species’ biology and pathogenicity. In order to gain further insight into the pathogenic potential of *D. haptotyla* YMF1.03409, based on the references to other existing literature (6), we selected 11 other fungi for comparative analysis alongside *D. haptotyla* YMF1.03409. These include: (i) four nematode-trapping fungi with different representative traps [*O. oligospora* and *A. flagrans* (adhesive networks), Drechslerella brochopaga (constricting ring), and *Dactylellina cionopaga* (unstalked adhesive knobs and two-dimension networks)]; (ii) five pathogenic fungi with an infecting effect on plants (*Pyricularia oryzae*, *Metarhizium anisopliae*, *Beauveria bassiana*, *Alternaria alternata*, and *Fusarium graminearum*); and (iii) two non-pathogenic fungi (*Aspergillus nidulans* and *Neurospora crassa*). The glycoside hydrolases (GH family) of *D. haptotyla* YMF1.03409 are more (202) than those of *O. oligospora* (195), *A. flagrans* (170), and lower than those of *A. alternata* (260), *P. oryzae* (248), and *A. nidulans* (256), and the differences in the numbers of fungi reflect their respective needs for carbon harvesting (Table S4).

### Cryptic phylogenetic history of *D. haptotyla* YMF1.03409 gene families

**Fig 3:**
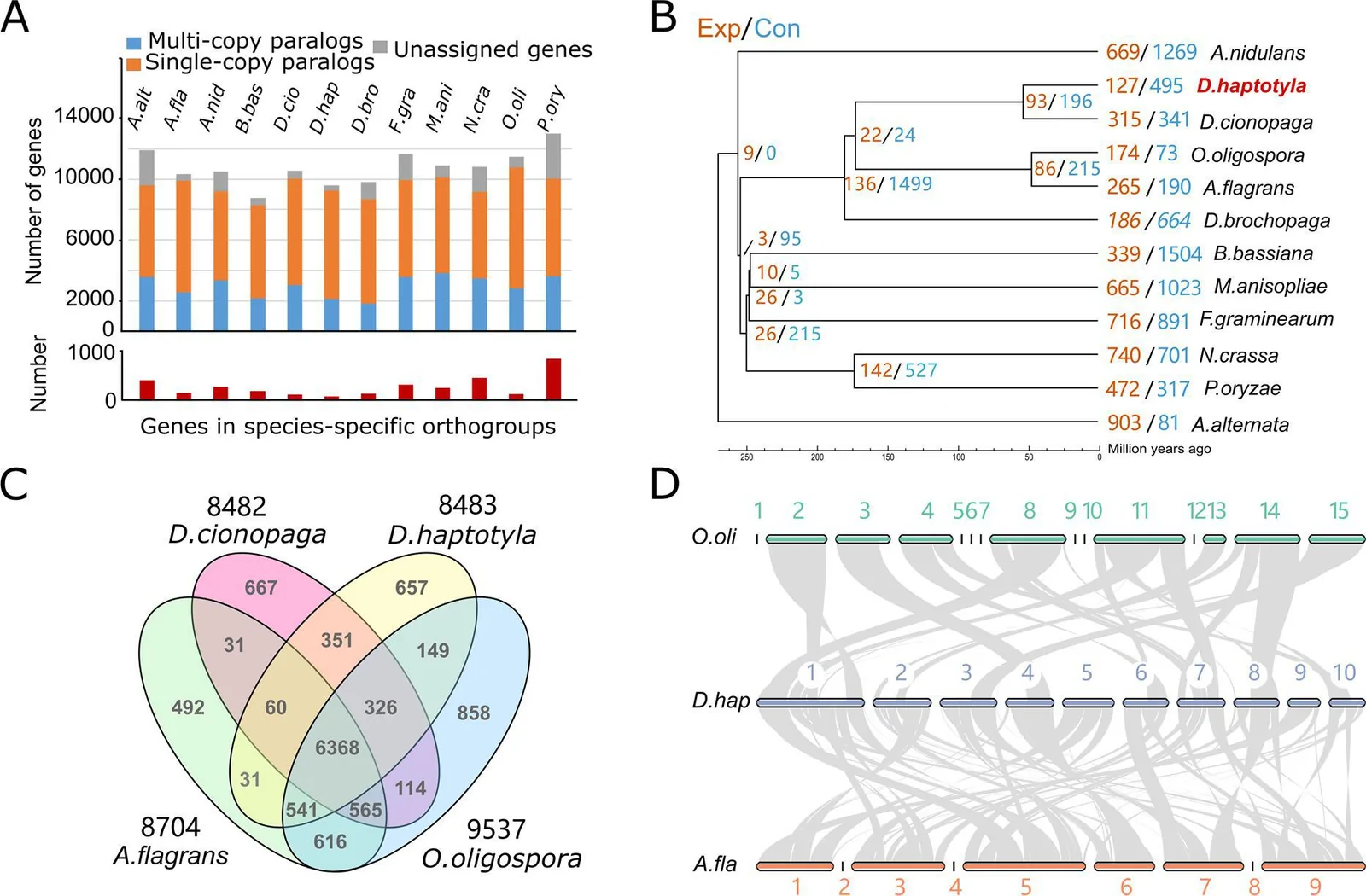
Comparative genomics of *D. haptotyla* YMF1.03409. (**A**) Statistics of genes in orthogroups among the 12 fungi. (**B**) The expanded and contracted gene families among the 12 fungi. (**C**) The overlapped and species-specific genes among *D. haptotyla* YMF1.03409 and three other nematode-trapping fungi. (**D**) Synteny map of *D. haptotyla* YMF1.03409 and two other nematode-predator fungi.

Eleven other sequenced fungal genomes were compared to the *D. haptotyla* YMF1.03409 genome. Among the 12 fungal genomes, we revealed 13,469 gene families and 115,022 genes, including 1,979 single-copy gene families; 3,306 gene families were shared by all 12 fungi. There are 24 gene families unique to *D. haptotyla* YMF1.03409, including 61 genes (Table S5). The clustering results are shown in Fig. 3A.

To investigate the evolutionary history of *D. haptotyla* YMF1.03409, we conducted a phylogenetic analysis. We selected 890 single-copy genes to construct a phylogenetic tree (Fig. S3). All five nematode-trapping fungi were located in the same branch, which is consistent with the results of previous studies (24, 33). Phylogenetic analysis by maximum likelihood was used to predict the divergence time, which showed that *D. haptotyla* YMF1.03409 diverged from other elaterids around 49.4 million years ago (Mya) (95% CI, 41.6–64.0 Mya) (Fig. S3). Based on the phylogenetic tree, we conducted the computational analysis of gene family evolution to predict the contraction and expansion of the gene families of the 12 fungi relative to their ancestors (Fig. 3B). A total of 247 gene families were changed in the branch leading to *O. oligospora*; 73 (29.6%) were contracted, and 174 (70.4%) were expanded. Among the 12 fungi, the *A. alternata* branch had the highest proportion of expanded gene families (91.8%). In the branch leading to *D. haptotyla* YMF1.03409, 622 gene families changed in size. Among these, 495 (79.6%) and 127 (20.4%) gene families were contracted and expanded, respectively. The contracted genes of *D. haptotyla* YMF1.03409 were enriched in terpenoid backbone biosynthesis (ko00900) and ATP-binding cassette transporters (ko02010), whereas the expanded genes were enriched in fructose and mannose (ko00051) and cysteine and methionine metabolism (ko00270) (Table S6). The contraction of a large number of gene families in *D. haptotyla* YMF1.03409 indicates that in the process of evolution, some useless genes were discarded to better adapt to the environment.

The analysis and comparison of the gene families of *D. haptotyla* YMF1.03409 with the other three nematode-trapping fungi (Fig. 3C) revealed that the *D. haptotyla* YMF1.03409 genome shared 149, 351, and 31 gene families with the *O. oligospora* genome, *D. cionopaga* genome, and *A. flagrans* genome, respectively. This suggests that the gene families of *D. haptotyla* YMF1.03409 and *D. cionopaga* were more similar in structure and function than those of the other three nematode-predatory fungi, indicating a closer relationship. There were 657 gene families unique to *D. haptotyla* YMF1.03409, compared to 492, 667, and 858 gene families unique to *A. flagrans*, *D. cionopaga*, and *O. oligospora*, respectively. The unique gene families of *D. haptotyla* YMF1.03409 were analyzed using gene ontology (GO) and Kyoto Encyclopedia of Genes and Genomes (KEGG) analyses. The GO enrichment results showed that these unique gene families were mainly concentrated on the cell surface and cell adhesion, which may be related to the formation of adhesion substances on the surface of *D. haptotyla* YMF1.03409 traps (Table S7). The KEGG enrichment results showed that these unique gene families were mainly involved in autophagy, biosynthesis of cofactors, and the mitogen-activated protein kinase (MAPK) signaling pathway (Table S7). The MAPK signaling pathway is important for pathogenic fungal signal transduction. It is a serine/threonine protein kinase that plays a pivotal role in cellular signaling by influencing gene transcription and regulation. It is involved in sexual mating, mycelial development, conidial peduncle formation, osmotic stress regulation, cell wall integrity, production and germination of conidia and adherent cells, and fungal pathogenicity (34). The collinearity synteny map also showed complex genomic translocation events among the different nematode-trapping fungi (Fig. 3D).

### BGCs identified in *D. haptotyla* YMF1.03409

**Fig 4:**
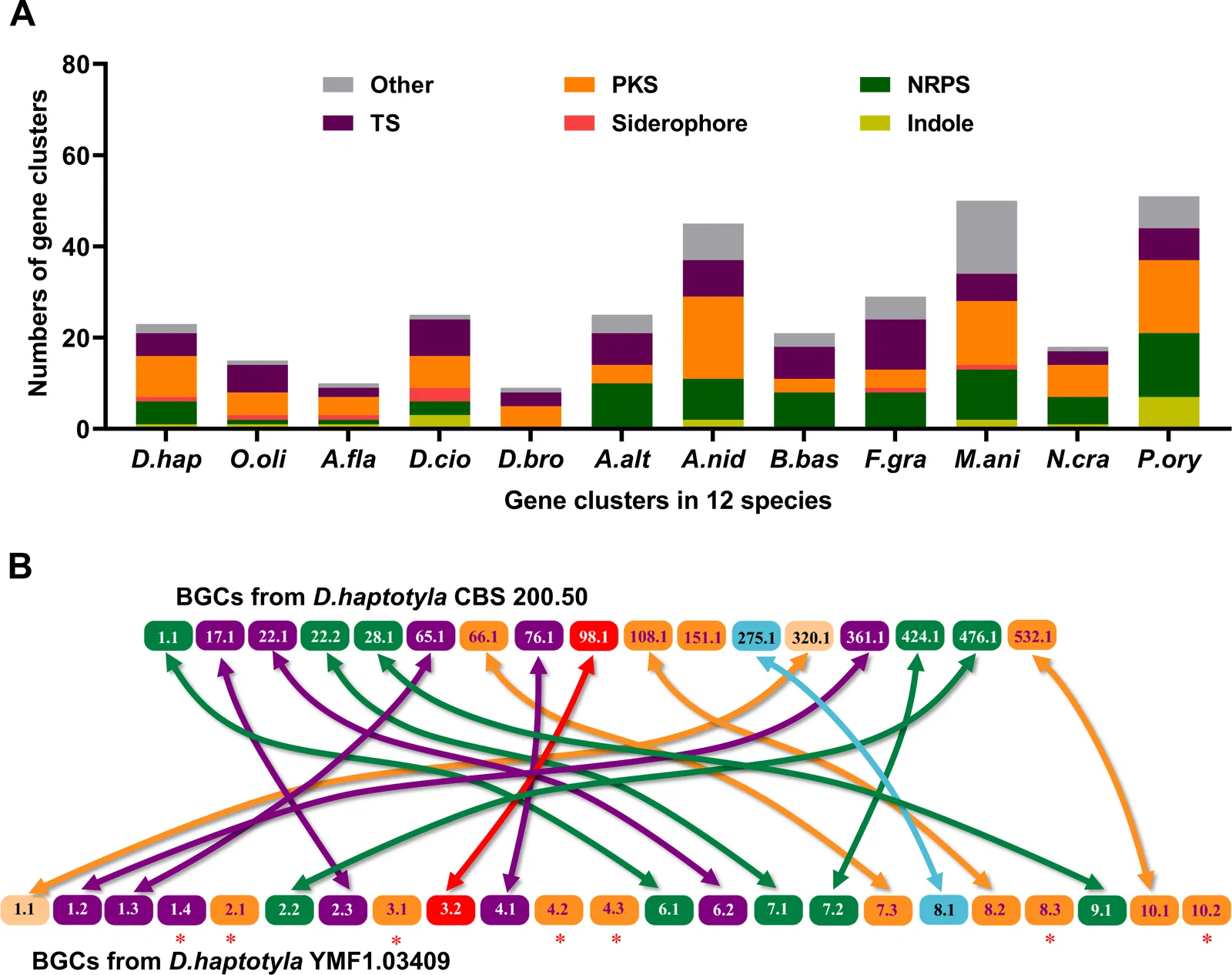
Analysis of BGCs of *D. haptotyla* YMF1.03409. (**A**) Statistics of BGCs among the 12 fungi. (**B**) Comparative BGCs between *D. haptotyla* YMF1.03409 and *D. haptotyla* CBS 200.50.

Filamentous fungi have the potential to produce a wide range of biologically active secondary metabolites that play an important role in infecting the host (35 – 37). To investigate the information related to the secondary metabolites of *D. haptotyla* YMF1.03409, we analyzed its genomes and those of 11 other fungal species using antiSMASH fungal version 6.1.1 (https://fungismash.secondarymetabolites.org/). In *D. haptotyla* YMF1.03409, antiSMASH analysis identified nine polyketide synthase (PKS) gene clusters, five non-ribosomal peptide synthetase (NRPS) gene clusters, five terpene gene clusters, and one NRPS-T1PKS gene cluster (Table S8). Notably, the size of the genome of *D. haptotyla* YMF 1.03409 is almost the same as that of ordinary filamentous fungi (Fig. 4A), but the BGCs comprise only approximately 50% of the general filamentous fungi. This suggests that the parasitic mechanism of the predator fungal genome leads to the replacement of synthetic secondary metabolite gene clusters that are not required for the parasitic process by amplified parasitic genes (27, 28, 38).

Furthermore, we compared and analyzed the BGCs from two genomes of the same species with different assembly methods; more secondary BGCs were identified from the chromosome-level assembly created by long-reading technology. According to the result, compared to the reported genome (GCA_000441935.1), the preset genome (JAQJAX000000000) contained seven more BGCs (1.4, 2.1, 3.1, 4.2, 4.3, 8.3, and 10.2) (Fig. 4B). The seven BGCs comprised one terpenoid BGC (1.4) and six PKS BGCs (2.1, 3.1, 4.2, 4.3, 8.3, and 10.2). One PKS BGC (151.1) in the previously assembled genome (GCA_000441935.1) was not fully matched in the newly assembled genome (JAQJAX000000000). Our analysis found that BGC 151.1 was incomplete, and the core protein contained only a ketide synthase domain (H072_4452), whereas the other protein (H072_4451) contained several domains (AT, DH, cMT, ER, and KR). The two main proteins (H072_4452 and H072_4451) in PKS BGC 151.1 showed certain similarities with the amino acid sequences of 2.1, 3.1, 4.2, and 4.3, respectively, in the assembled genome (JAQJAX000000000). This is consistent with our earlier failure to clone the core gene of cluster 151.1 from the genome. We used the genomic data (GCA_000441935.1) of *D. haptotyla* CBS 200.50 as a reference, but PCR amplification failed to amplify the band of interest, and the multi-segment sequence of cluster 151.1 was amplified and sequenced, we discovered that this gene cluster’s multiple fragment sequencing information was missing. These findings show that chromosome-level deep-coverage sequencing can speed up the investigation of BGCs in *D. haptotyla*.

### Transcriptional responses during nematode trap formation

**Fig 5:**
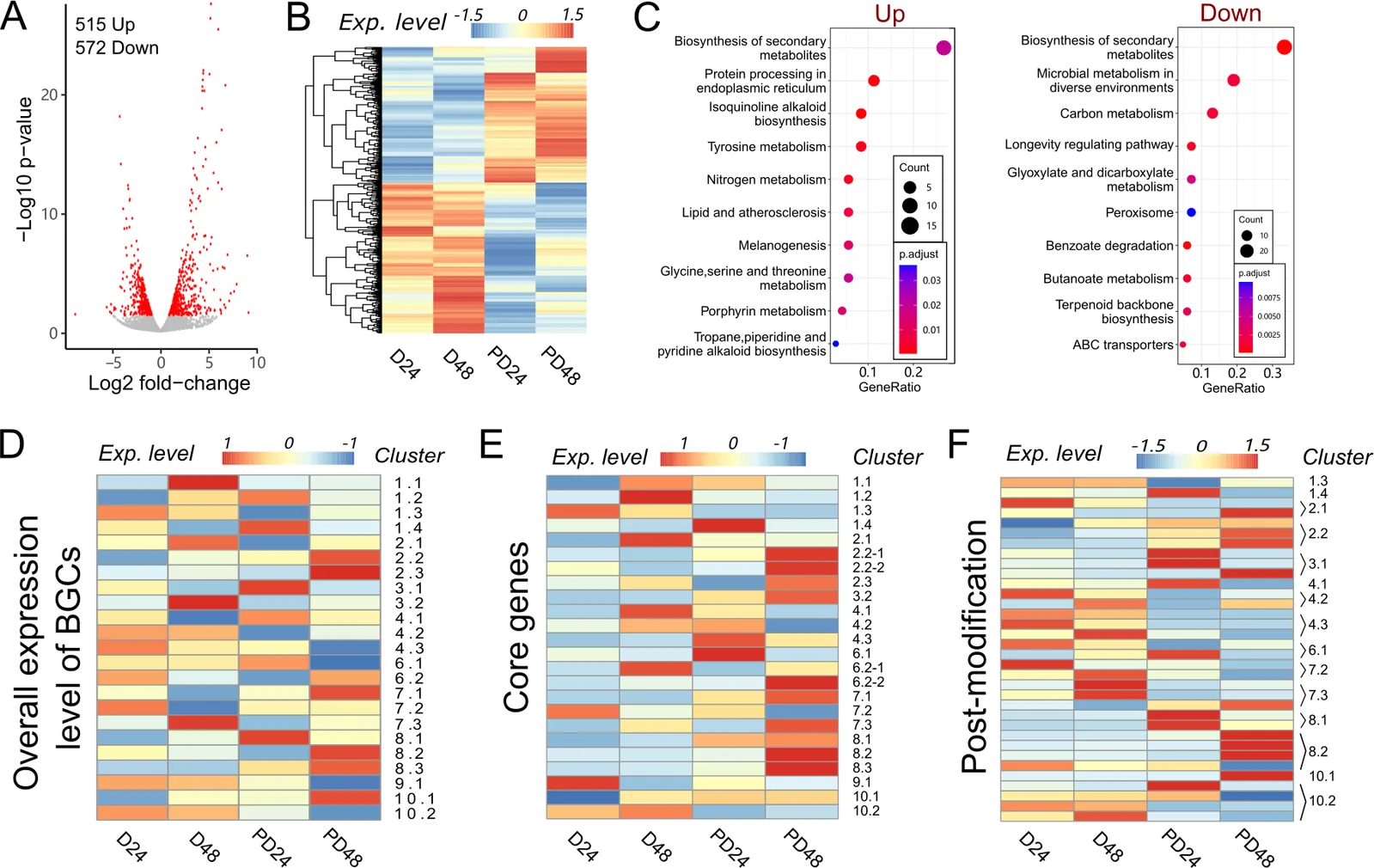
The transcriptome analysis of *D. haptotyla* YMF1.03409 gene transcription dynamics of the nematodes-infection assay. (**A**) The volcano plot of DEGs. The red color represents DEGs with significance (*P* < 0.05). The *x*-axis represents the expression change level of PD group relative to D group, and *y*-axis represents the significant level. (**B**) The heatmap of the expression levels of 1,087 DEGs. (**C**) The KEGG pathway enrichment results of upregulated (left) and downregulated (right) genes. The heatmaps of (**D**) 23 secondary metabolite biosynthetic gene clusters, (**E**) core genes of gene clusters, and (**F**) post-modification genes of gene clusters.

We further assessed the dynamic changes in gene expression during nematode infection. We conducted RNA-seq on *D. haptotyla* YMF1.03409, which was induced by *P. redivivus* for different periods. Among the 11,073 genes, 515 and 572 genes were significantly upregulated and downregulated under nematode infection status, respectively (Fig. 5A; Table S9). The shikimic acid pathway plays a central role in the secondary metabolic pathways of a broad range of organisms, including plants, fungi, bacteria, and some protozoans (39). Among the DEGs that were significantly upregulated, we identified a gene associated with the shikimic acid pathway (*EVM08G003990.1*), suggesting that its associated metabolites may be related to the infection of nematodes by *D. haptotyla* YMF 1.03409 (Fig. S4). The heatmap shows that these differences in DEG expression were obvious in the control and infection groups (Fig. 5B). In the KEGG pathway enrichment analysis, 13 pathways were significantly enriched in upregulated genes (Fig. 5C; Table S10), including the biosynthesis of secondary metabolites (ko01110) and nitrogen metabolism (ko00910). A series of metabolic pathways were also enriched, such as the MAPK signaling pathway (ko04016), and lipid and atherosclerosis (ko05417). Among the downregulated genes, 21 pathways were significantly enriched (Fig. 5C; Table S10), including benzoate degradation (ko00362), carbon metabolism (ko01200), inositol phosphate metabolism (ko00562), and terpenoid backbone biosynthesis (ko00900).

As described in the previous section, the *D. haptotyla* YMF1.03409 genome includes 23 secondary metabolite biosynthetic gene clusters. The expression levels of these 23 secondary metabolite gene clusters and genes within the clusters were examined, and it was discovered that the majority of secondary metabolite gene clusters were elevated during infection in comparison to the control group. The expression levels of eight secondary metabolite gene clusters showed significantly upregulated expression at 24 h after nematode addition, and 11 secondary metabolite biosynthetic gene clusters showed significantly upregulated expression at 48 h (Fig. 5D). To further understand the changes in the expression levels of functional genes in gene clusters during the infection process, we analyzed the expression levels of core and post-modification genes in the secondary metabolite gene clusters. Among these, post-modification genes (additional biosynthetic genes) have an important modifying role in the structure formation of the corresponding product of gene cluster. The expression level of each cluster was estimated by singular value decomposition, which was implemented by pathway level analysis of gene expression algorithm (40). The analysis results suggest that the expression levels of core genes of 11 and 12 gene clusters were upregulated at 24 and 48 h after infestation, 4 and 10 of which were significantly upregulated, respectively (Fig. 5E). The expression levels of post-modification genes also exhibited significant changes at different time points (Fig. 5F). These results imply that secondary metabolites may be crucial to *D. haptotyla* YMF1.03409’s ability to infect nematodes. We also found that a fraction of DEGs matched with PHI-base and P450-base entries (Fig. S5; Table S11).

The gene expression level may fluctuate with the nematode infection time. Subsequently, we estimated the time-series changes of DEGs at 24 and 48 h. Among the 1,087 DEGs, we identified four consistent gene groups using the fuzzy-cluster method (Fig. S6). Genes in the four groups were also enriched in various KEGG terms (Fig. S6; Table S12).

### Metabolomics analysis of *P. redivivus* infection by *D. haptotyla* YMF1.03409

**Fig 6:**
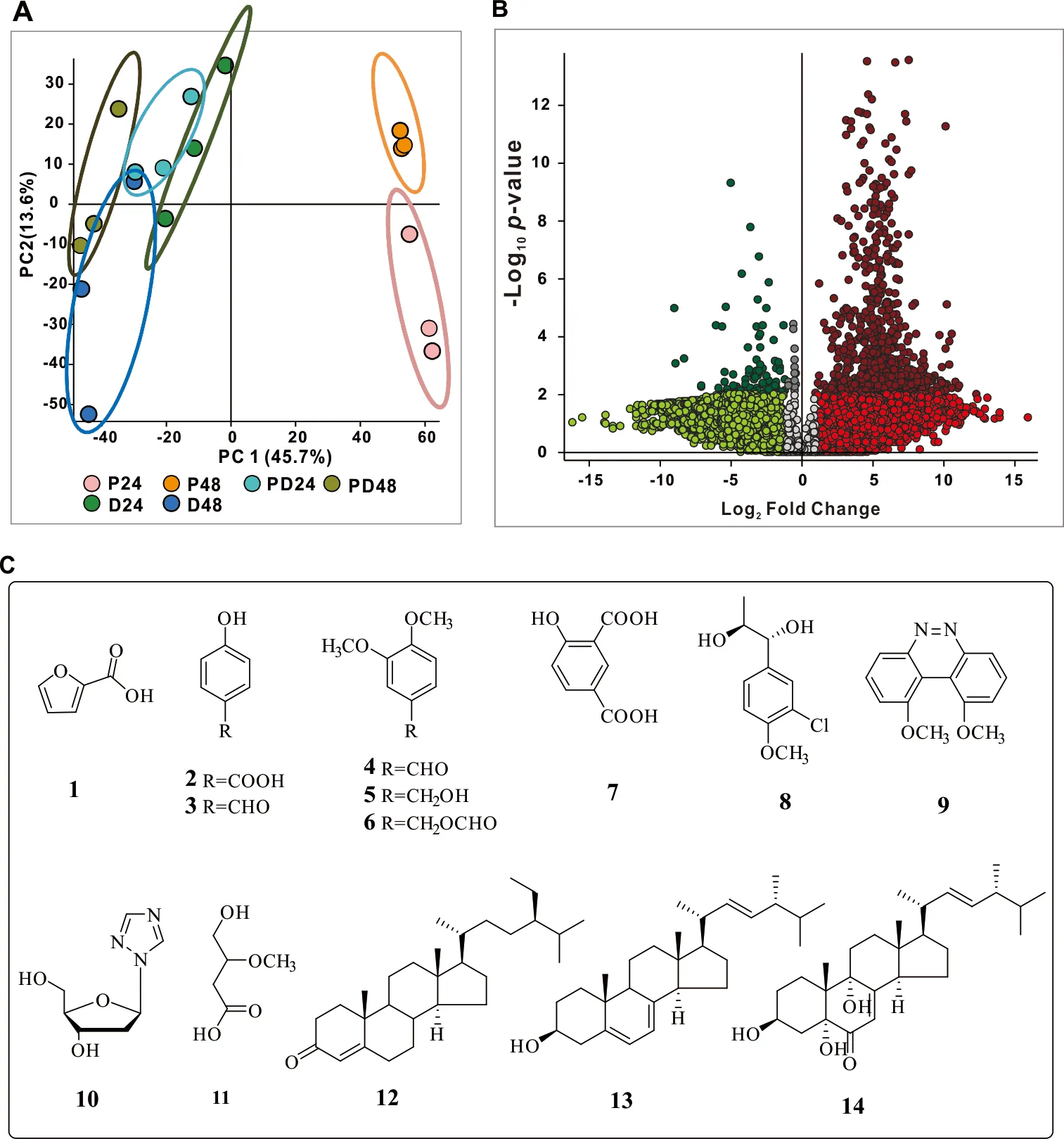
The metabolome analysis during *P. redivivus* infection by *D. haptotyla* YMF1.03409 and that of the metabolites obtained from the fermentation of *D. haptotyla* YMF 1.03409. (**A**) PCA of all extracts. (**B**) The volcano plot of PD48 vs D48 group. Significance cutoffs were *P* = 0.01 (Bayes moderated *t*-tests) and fold change (FC) = 1. Each dot represents an individual compound (within ±10 ppm in mass), and the probability of that quantitative observation being statistically significant is indicated by a *P* value on the *y*-axis (determined using the standard linear model within the SIEVE software). (**C**) The structures of metabolites from the fermentation of *D. haptotyla* YMF1.03409.

To determine whether metabolites and metabolic pathways were altered during *P. redivivus* infection by *D. haptotyla* YMF1.03409, the extract samples were subjected to LC-MS of untargeted metabolomics. The quantitative analysis of low-molecular-weight metabolites can reveal the relative relationship between changes and metabolites and may indicate metabolite dynamics during the infection of *P. redivivus* by *D. haptotyla* YMF1.03409. Extracted metabolites were analyzed in the positive ion mode as described in the Materials and Methods section. Loading data for the principal component analysis (PCA) were derived from all metabolites identified by Compound Discoverer 3.3 after the LC-MS analysis and their peak area tendencies. The first and second principal components (PC1 and PC2, respectively) accounted for 59.3% of the overall variance. The P24 and P48 groups were significantly separated from all the other groups in PC1 (45.7%) (Fig. 6A). The PD48 group was clearly separated from the D24 and D48 groups. This result highlights the significant differences in the major metabolites produced by *D. haptotyla* YMF1.03409 during its infection of *P. redivivus*. Therefore, based on the PCA results and experimental purposes, we focused on metabolite changes between the PD and D groups. By combining all the analyzed extracts, unique molecular species were detected by UPLC-HR-ESI-MS. The high-resolution MS signals from different isotopes and adduct peaks were combined to ensure that the vast majority of molecular species represented the individual metabolites produced by the corresponding strain. We aimed to determine the differences in secondary metabolites between the PD and D groups, and the data were displayed as a volcano plot. Using significance cutoffs of a false discovery rate-adjusted *P-*value (<0.01) and a fold-change difference >1, we observed that 71 metabolites were upregulated in PD24 vs D24, 67 metabolites were downregulated in PD24 vs D24 (Fig. S7), 789 metabolites were upregulated in PD48 vs D48, and 117 were downregulated in PD48 vs D48 (Fig. 6B). To determine the structures of the upregulated compounds, we constructed a structural library containing 249 metabolites from nematode-trapping fungi.

The complete masses of the upregulated compounds were used to search the structural library and other libraries (MZCloud, ChemSpider, and MZVault). When searching these databases with a mass tolerance of 10 ppm, 28 annotations were verified and are listed in Table S13. The 28 identified compounds included various structural types, such as NRPS, PKS, and terpenes. Subsequently, we quantitatively analyzed these and other metabolites in response to the PD24 and PD48 groups. Infection of *P. redivivus* by *D. haptotyla* YMF1.03409 led to a sixfold increase in metabolite production of >96% of known compounds in PD48 compared to the D48 (wild-type) samples (Table S13); a change greater than 20-fold was observed for >85.7% of known metabolites relative to the D48 samples. Among them, the relative contents of two metabolites (2-furoic acid and paganin B) increased by 750- and 867-fold, respectively (Table S13). In the next experiment, we fermented *D. haptotyla* YMF1.03409 to obtain these metabolites and investigated their function during the infection of *P. redivivus* by *D. haptotyla* YMF1.03409.

The crude extract (35.64 g) produced by the fermentation of *D. haptotyla* YMF1.03409 was isolated and purified, and 14 metabolites were extracted (Fig. 6C). All compounds were identified by MS and nuclear magnetic resonance data (the spectral data are presented in the supplemental material). Four of them were identified from the fermentation product and annotated in the metabolome of *P. redivivus’*s infection by *D. haptotyla* YMF1.03409, and the relative peak area differences were displayed in the LC-MS chromatograms (Fig. S8).

### Nematicidal activity of 2-furoic acid confirmed by virulence assay

**Fig 7:**
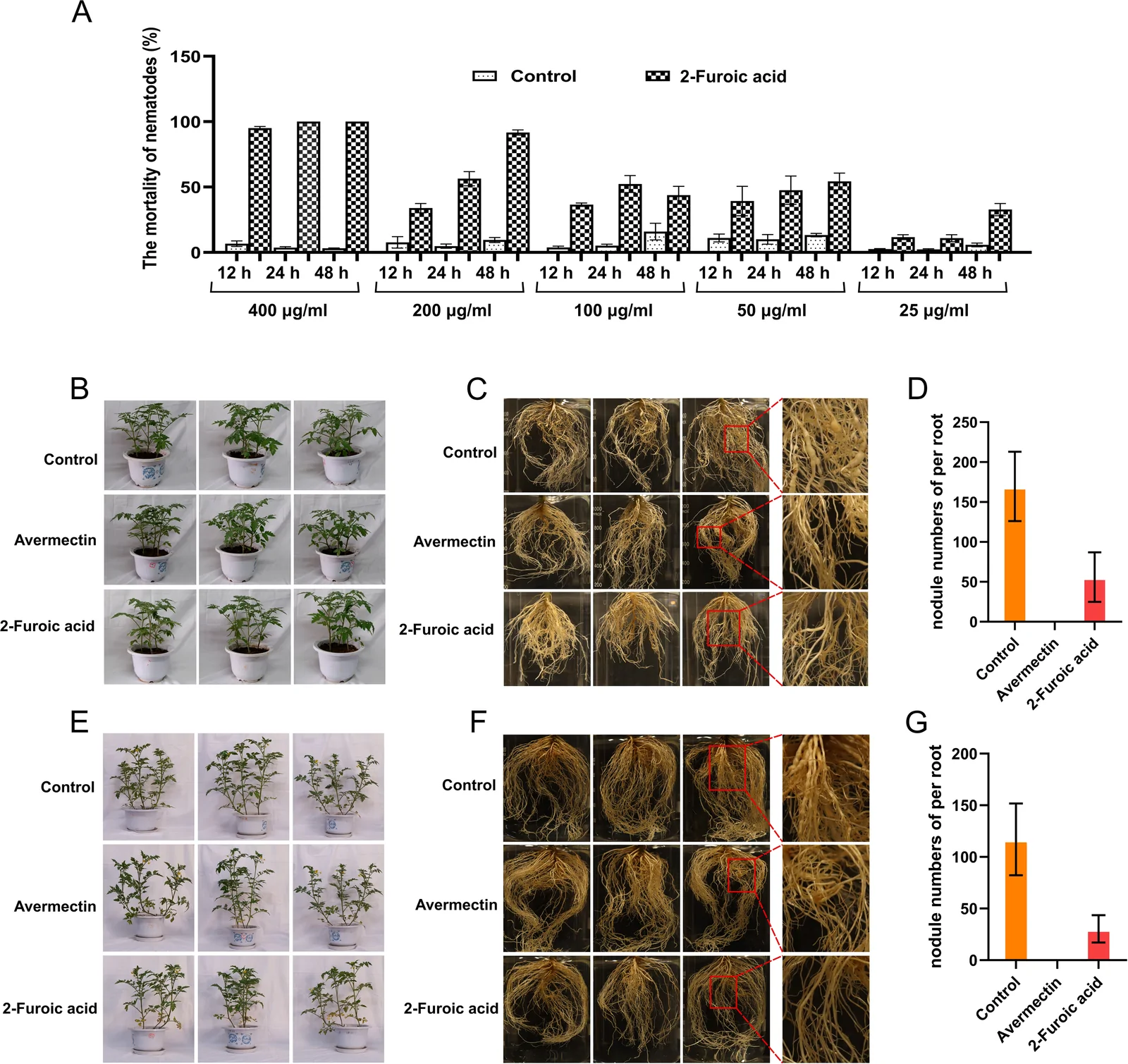
Nematicidal and anti-infestation ability of 2-furoic acid. (**A**) Lethality of different concentrations of 2-furoic acid on *M. incognita*. (**B**) Tomato growth status in the experimental and control groups after 20 days; control represents the negative control and avermectin represents the positive control. (**C**) State of infestation of the roots of the experimental and control groups after 20 days. (**D**) Statistics on the number of galls in the root system of experimental and control groups after 20 days. (**E**) Tomato growth status in the experimental and control groups after 35 days; control represents the negative control, avermectin represents the positive control. (**F**) State of infestation of the roots of the experimental and control groups after 35 days. (**G**) Statistics on the number of galls in the root system of experimental and control groups after 35 days.

The test concentrations utilized in the nematicidal activity assay for *M. incognita* were 400, 200, 100, 50, and 25 µg/mL, and the results showed that 2-furoic acid (1) had excellent nematicidal activity. At 48 h, the mortality rate of *M. incognita* was greater than 90% at 2-furoic acid concentrations of 400 and 200 µg/mL. At a concentration of 50 µg/mL, the lethality rate of *M. incognita* reached 50% at 48 h. The LD_50_ value of 2-furoic acid for *M. incognita* at 48 h was 55.05 µg/mL (Fig. 7A). We also evaluated 2-furoic acid’s lethality on *P. redivivus* and *C. elegans*; it has also shown great nematicidal ability, and after 48 h, its respective LD_50_ values were 3.33 and 147.63 µg/mL (Fig. S9). The excellent nematicidal ability of 2-furoic acid and its general insecticidal effect indicate its potential in the development of biological control agents. 2-Furoic acid is a heterocyclic carboxylic acid that has been widely used in the pharmaceutical, agrochemical, flavor, and fragrance industries (41). In the industry, furfural is obtained by the depolymerization of xylose in hemicellulose, and xylose is produced after acid-catalyzed dehydration (42). Furfural is then catalyzed by aldehyde dehydrogenase to produce 2-furoic acid (43). In 2022, Wang et al. reported that 2-furoic acid obtained from *Aspergillus fumigatus* 1 T-2 had strong toxic activity against *Meloidogyne incognita* (44). However, how 2-furoic acid is synthesized in microorganisms, especially fungi, still remains unclear. Based on the present data, we speculated that the biosynthesis of 2-furoic acid in *D. haptotyla* YMF 1.03409 is not a derivative of xylan degradation products. The most critical reason for this is that we did not detect furfural, which is the key degradation product of xylan, in all control samples. Additionally, the metabolomics data indicate that the relative content of 2-furoic acid is significantly increased in the PD group after 48 h of infection of nematodes by *D. haptotyla* YMF1.03409; after 24 h, although 2-furoic acid could be detected, its relative content was very low. Therefore, we believe that there is an undiscovered pathway of 2-furoic acid synthesis in *D. haptotyla* YMF1.03409; we preliminarily speculated the occurrence of the following two pathways: some microorganisms can produce key intermediate products of furanolactone rings by degrading benzene rings and further modifying them to form 2-furoic acid, some of which have been found in bacteria (45). Moreover, six-carbon units can be synthesized from three acetic acid units through the polyketide pathway, and subsequently, post-modification can finally form 2-furoic acid. Both these pathways require extensive experimentation.

Additionally, we tested the nematicidal activity of the remaining compounds, of which 4-hydroxybenzoic acid (2) and 4-hydroxybenzaldehyde (3) showed nematicidal activity (Fig. S10).

### Furoic acid repressed the root-knot nematode infestation

Twenty days after planting the tomato seedlings, we observed and evaluated the growth of the tomatoes as well as the development of root-knot nematode infestation. The growth status of tomatoes indicated no significant difference between the experimental and control groups (Fig. 7B), suggesting that 2-furoic acid has no negative effect on the growth of tomatoes. The experimental group had an average of 50 galls per tomato root and a root-knot index of 63%, whereas the negative control group had an average of 170 galls per tomato root and a root-knot index of 95% (Fig. 7C and D). At an infestation time of 35 days, 2-furoic acid still showed better resistance to infestation. Similarly, no significant difference was observed between the experimental and control groups in terms of the plant growth status (Fig. 7E). In terms of the degree of root infestation, the experimental group had an average of 30 galls per tomato root and a root-knot index of 62%, while the negative control group had an average of 121 galls per tomato root and a root-knot index of 90% (Fig. 7F and G).

## DISCUSSION

More than 380 taxonomically different fungi have been identified as nematode-trapping fungi (46), all of which may infect and kill worms. Nematode-trapping fungi enter the parasitic stage via the product of specialized morphological tissues and organs known as traps (mostly constricting rings, adhesive networks, adhesive columns, and adhesive knobs, among others), capturing and digesting nematodes. Because of this effective mechanism, a portion of the fungi has been commercially employed as an antinematode agent (47, 48). In recent years, several studies have been conducted on the genome, transcriptome, and proteome of nematode-trapping fungi, which indicate possible genomic evolutionary adaptation mechanisms enabling these predator fungi to acquire a parasitic lifestyle; namely, the formation of new genes through gene replication, including gene family amplification and differential gene expression of orthologous genes. The function and expression patterns of these genes have evolved in response to their interactions with nematode hosts (6, 28, 49). Notably, the majority of these nematode-trapping fungi genomes are roughly the same size as the genomes of typical filamentous fungi, but the BGC numbers are only about half as high. Therefore, we believe that the parasitic mechanism of the nematode-trapping fungi genome leads to the amplification of parasitic genes replacing the BGCs that are not required for the parasitic process, and the various metabolite BGCs remaining in the genome play an important role in the infection process (28, 38, 49). Through the analysis of the genomes of nematode-trapping fungi, we found that the genomes still contained a considerable number of BGCs (Fig. 4A; Table S8) (38). Moreover, the expression of BGC genes was significantly enhanced in the predation process (5, 26). Filamentous fungi generally have the potential to produce a wide range of biologically active secondary metabolites, and many pathogenic fungi produce metabolites that are often causative factors playing an important role in infecting the host (35). The enzymes that synthesize these metabolites have been formed by evolutionary mechanisms over thousands of years to ensure that the microorganisms that produce these metabolites maintain their environmental niche; however, a large proportion of fungal metabolites participating in infection remains undiscovered (50, 51).

*D. haptotyla* is one of the representative species for studying the mechanism of the interaction between nematode-trapping fungi and nematodes, which capture nematodes by generating a specific single-cell structure known as knobs. Current *D. haptotyla* research has primarily focused on the physiological mechanisms of infestation, with no in-depth metabolite investigations described. Furthermore, due to the limitations of the sequencing level at the time, as well as the quality and representativeness of its genomic data, the published information on the genome of *D. haptotyla* is insufficient for our investigation. Here, we created the first chromosomal-level assembly of the *D. haptotyla* YMF1.03409 genome and annotated its genetic makeup in great detail. In this study, we compared the genome of *D. haptotyla* YMF1.03409 with those of 11 other fungal species. The results of the gene family analysis showed that *D. haptotyla* YMF1.03409 and *D. cionopaga* shared a greater number of gene families, suggesting that their evolutionary relationships are closer relative to other nematode predatory fungi, and this is also consistent with the results of the phylogenetic tree. CAZYs are thought to be associated with nutrient perception and acquisition and are involved in determining the saprophytic capacity of the fungus. Analysis of the CAZY database revealed that the number of glycoside hydrolases (202) in the *D. haptotyla* YMF1.03409 genome is comparable to that of *O. oligospora*, more than that of *A. flagrans* and less than those of *A. alternata* and *A. nidulans*. These results illustrate their saprophytic abilities. *A. flagrans* contains fewer GH families, indicating a weaker saprophytic capacity, whereas similar numbers of GH families in *D. haptotyla* YMF1.03409 and *O. oligospora* indicate that they are closer in terms of carbon source acquisition. Additionally, the number of genes associated with pathogenicity in the *D. haptotyla* YMF1.03409 genome was large compared to that in the PHI-base, indicating its potentially strong pathogenicity. Small-molecule compounds play an important role in fungal-host interactions. A comparison of the metabolite gene cluster information between *D. haptotyla* YMF1.03409 and other fungi revealed that *D. haptotyla* YMF 1.03409 contains a smaller number of gene clusters with similar genome sizes, suggesting evolutionary adaptation to the parasitic state.

Second, to analyze the differential expression of genes associated with the infection process, we also investigated the transcriptional expression levels of various genes and metabolic profiles during the infection process using transcriptomics and metabolomics. We identified a small molecule compound, 2-furoic acid, which appeared specifically in reciprocal metabolic samples by combining genomics, transcriptomics, and metabolomics. Additionally, a gene in the shikimic acid pathway was associated with 2-furoic acid synthesis, and its expression level was also significantly upregulated during infestation. 2-Furoic acid was purified from the fermentation product of *D. haptotyla* YMF1.03409. Tests on the nematicidal activity and anti-infestation activity revealed excellent nematicidal ability and illustrated the great potential of 2-furoic acid for the development of biological control formulations. In summary, we propose a model for the action of 2-furoic acid during infection by *D. haptotyla* YMF1.03409. At the early stage of infection, *D. haptotyla* YMF1.03409 senses the presence of nematodes and produces a large number of knobs induced by nematodes. Meanwhile, the expression levels of genes related to 2-furoic acid synthesis in *D. haptotyla* YMF1.03409, such as *EVM08G003990.1* or PKS gene cluster, were significantly upregulated, prompting the production of 2-furoic acid in large quantities. 2-Furoic acid, as an active molecule with strong nematicidal activity against nematodes, poisoned the nematodes during infection and greatly increased the efficiency of *D. haptotyla* YMF1.03409 in capturing nematodes.

For the first time, we propose that 2-furoic acid plays a major role in the infection process of *D. haptotyla* YMF1.03409. These findings also imply that in-depth research on the mechanism of fungal-nematode interactions from the standpoint of small-molecule metabolites, such as the synthesis pathways of functional small-molecule compounds and the construction of engineered strains, will have a significant impact on nematode biocontrol.

## Data Availability

This whole-genome sequencing project has been deposited at DDBJ/ENA/GenBank under the accession number JAQJAX000000000.

## SUPPLEMENTAL MATERIAL

Supplemental file 1 spectrum.01896-23-s0001.pdf Fig. S1 to S10; Tables S1, S2, S6, S8 and S13; Methods S1 to S7; and Data S1

Supplemental file 2 spectrum.01896-23-s0002.xlsx Tables S3, S5, S7, and S9 to S12

Supplemental file 3 spectrum.01896-23-s0003.xlsx Table S4

ASM does not own the copyrights to Supplemental Material that may be linked to, or accessed through, an article. The authors have granted ASM a non-exclusive, world-wide license to publish the Supplemental Material files. Please contact the corresponding author directly for reuse.
