# Supplementary material for: 2-Furoic acid associated with the infection of nematodes by Dactylellina haptotyla and its biocontrol potential on plant root-knot nematodes
Source: Microbiol Spectr. 2023 Sep 27;11(5):e01896-23. doi: 10.1128/spectrum.01896-23 (PMC10580851; doi:10.1128/spectrum.01896-23)
Supplement: Supplemental file 1 — Fig. S1 to S10; Tables S1, S2, S6, S8 and S13; Methods S1 to S7; and Data S1 [file spectrum.01896-23-s0001.pdf]

Microbiology Spectrum **Supporting Information**

2-Furoic acid associated with the infection of nematodes by *Dactylellina haptotyla* and its biocontrol potential on plant root-knot nematodes

Hong-Mei Lei, Jun-Tao Wang, Qian-Yi Hu, Chun-Qiang Li, Ming-He Mo, Ke-Qin Zhang, Guo-Hong Li, Pei-Ji Zhao*

State key Laboratory for Conservation and Utilization of Bio-Resources in Yunnan, School of Life Sciences, Yunnan University, Kunming, Yunnan 650091, China.

*Correspondence and requests for materials should be addressed to Pei-Ji Zhao (email: pjzhao@ynu.edu.cn)

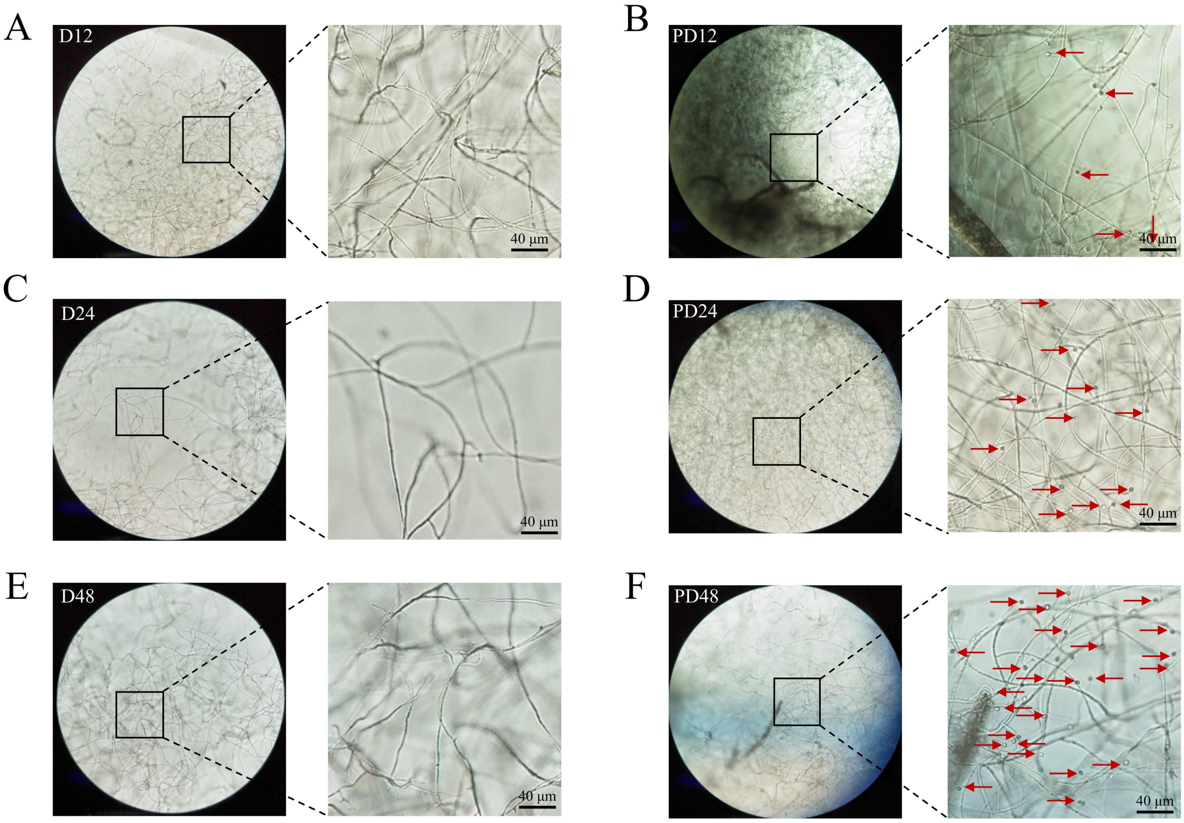

Figure S1. Infection status at different time points after the addition of nematodes. (A) Adhesive knobs in control group at 12 h after nematode addition. (B) Adhesive knobs in reciprocal group at 12 h after nematode addition. (C) Adhesive knobs in control group at 24 h after nematode addition. (D) Adhesive knobs in reciprocal group at 24 h after nematode addition. (E) Adhesive knobs in control group at 48 h after nematode addition. (F) Adhesive knobs in reciprocal group at 48 h after nematode addition. The adhesive knobs in the diagram are indicated by the red arrow.

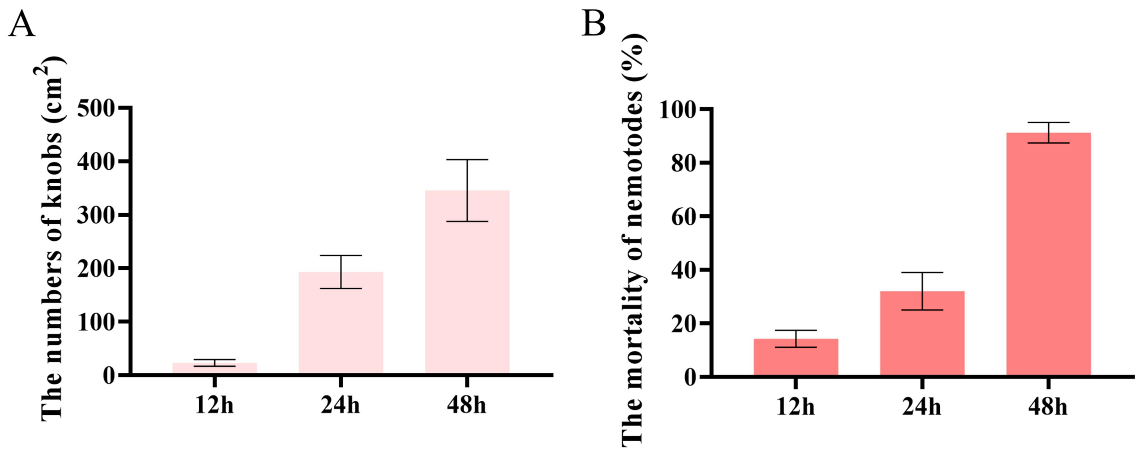

Figure S2. The pathogenicity of *D.haptotyla* YMF1.03409. (A) The number of adhesive knobs of *D.haptotyla* YMF1.03409 at 12, 24 and 48 h after challenge of *Paragrellus redivivus*. (B) The mortality rate of nematodes at 12, 24 and 48 h after challenge of *P. redivivus*.

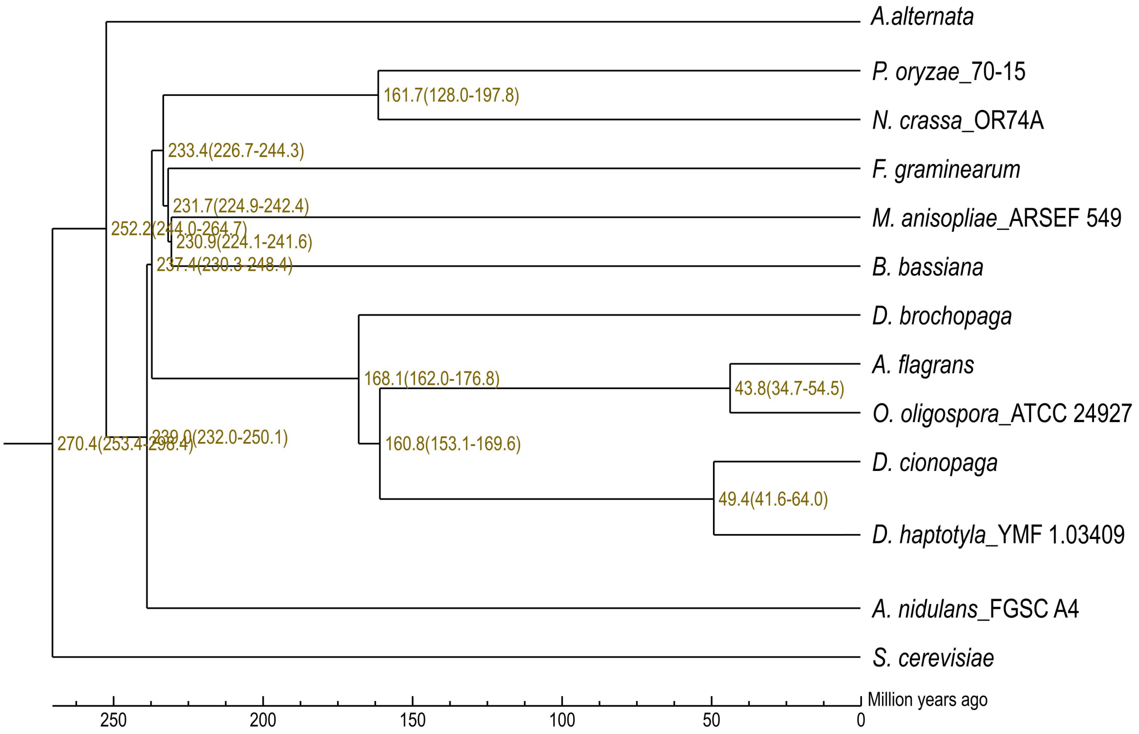

Figure S3. Divergence time of species. Inner nodes are labeled with the reference differentiation time, and the confidential intervals of differentiation time are in parentheses. The branch length indicates the length of divergence time.

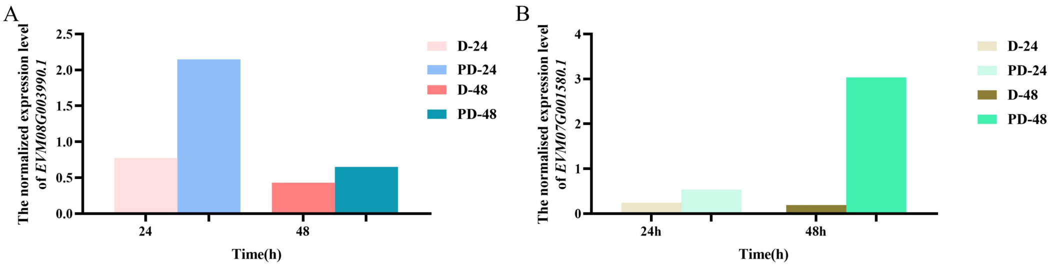

Figure S4. The normalized expression level of (A) *EVM08G003990.1* and (B) *EVM07G001-580.1*. *P*-value = 0.047 (*EVM08G003990.1*), *P*-value = 0.094 (*EVM07G001580.1*), which are calculated by DEseq2. *P*-values were calculated by comparing two controls (D-24 and D-48) and two infections (PD-24 and PD-48).

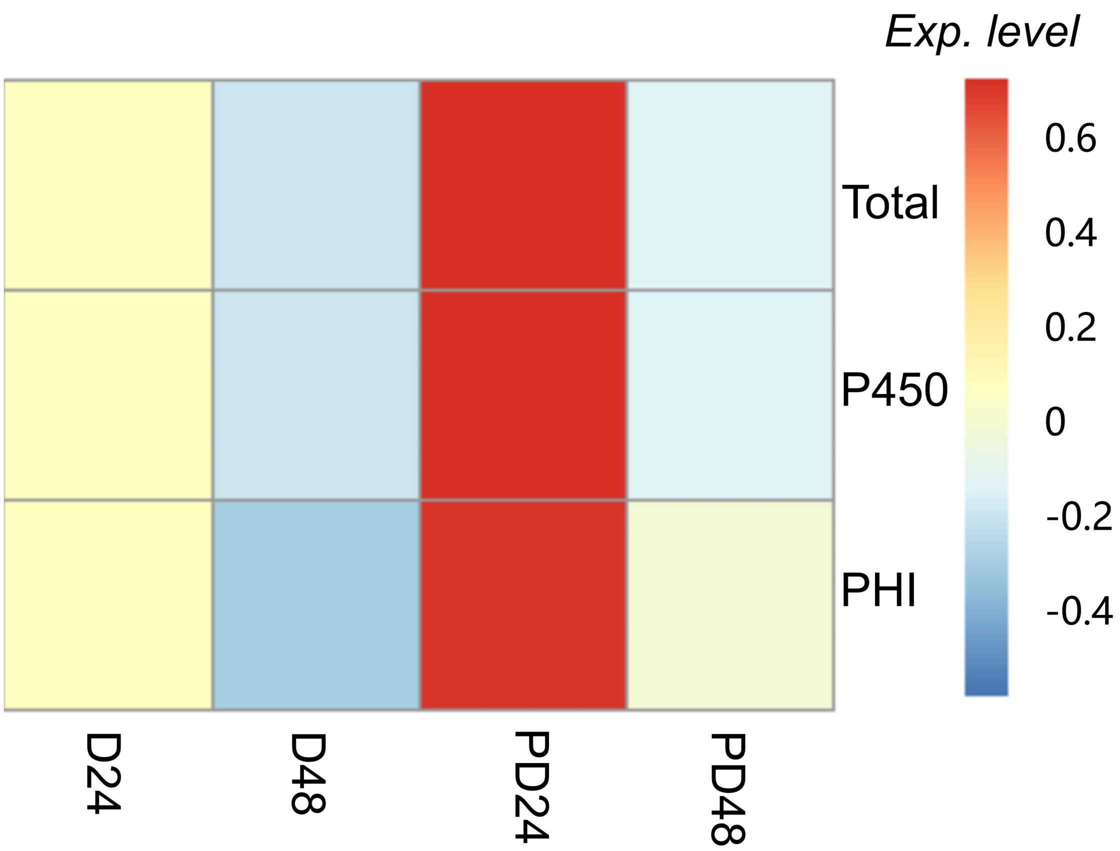

Figure S5. GSVA-estimated global expression level of P450 and PHI gene sets. “Total” represents the sum set of above 2 sets.

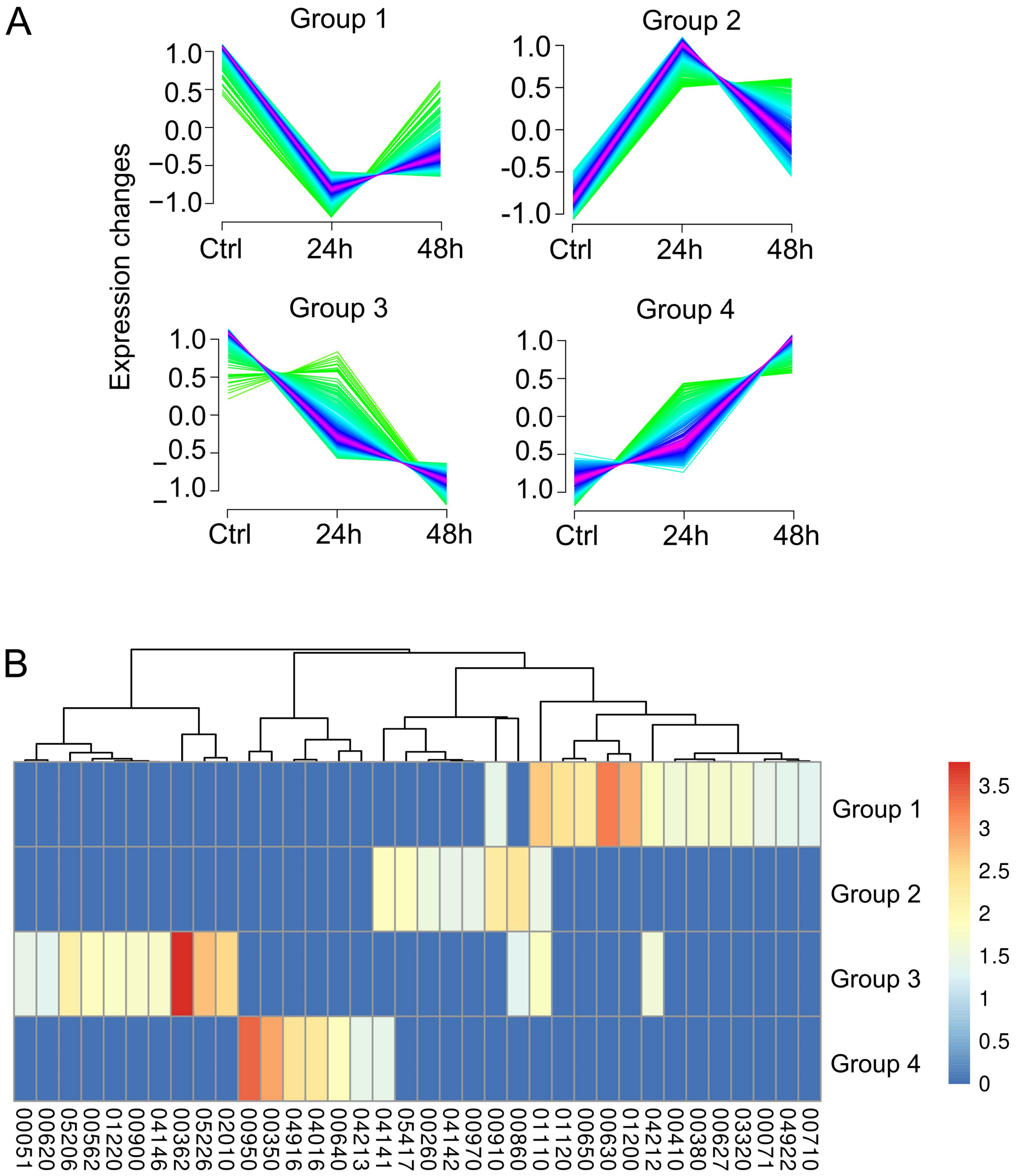

Figure S6. (A) Expression dynamic trajectories of DEGs fuzzy-clusters. There exist four consistent groups. (B) The KEGG pathway enrichment matrix of genes from four groups.

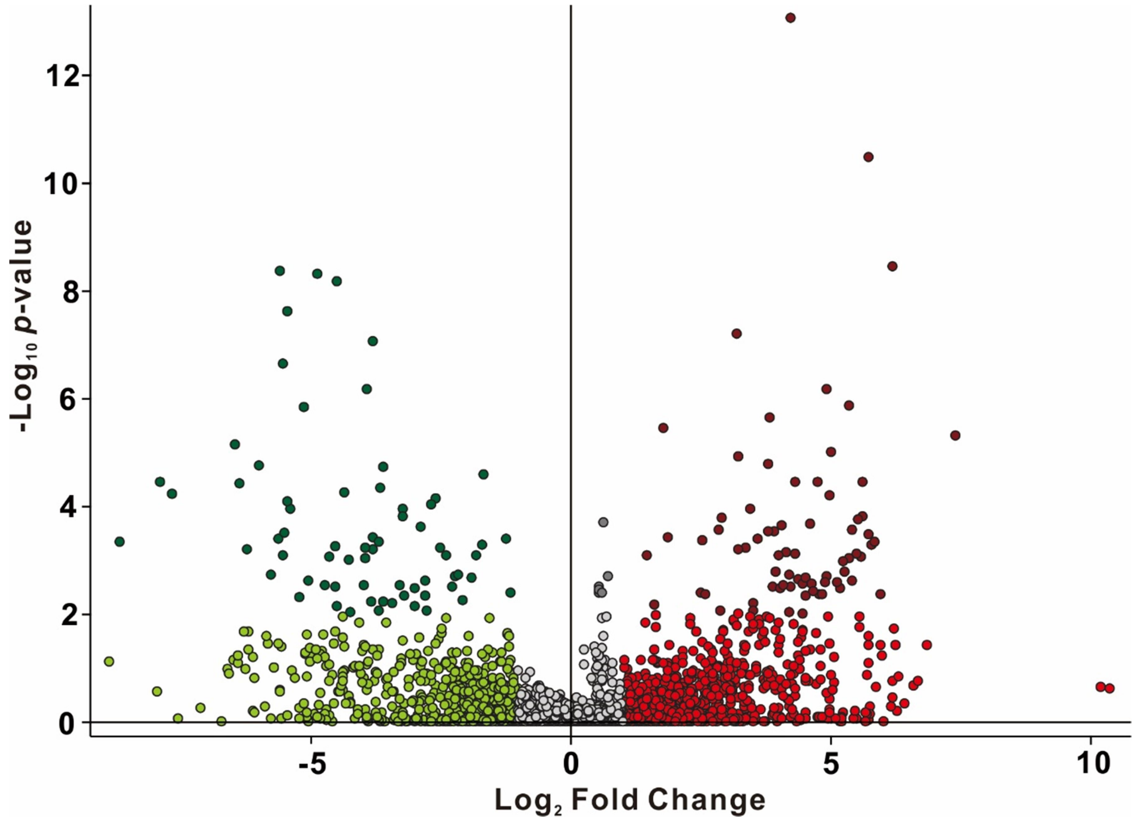

Figure S7. The volcano plot of PD24 versus D24 group. Significance cutoffs were $p = 0.01$ (Bayes moderated t-tests) and FC = 1. Each dot represents an individual compound (within ±10 ppm in mass), and the probability of that quantitative observation being statistically significant is indicated by a p value on the y-axis (determined using the standard linear model within SIEVE software). The 71 compounds on the right half of the plot are present at significantly higher levels in the PD24 samples. The 67 compounds on the left-hand portion of the diagram are present at significantly higher levels in the D24 samples.

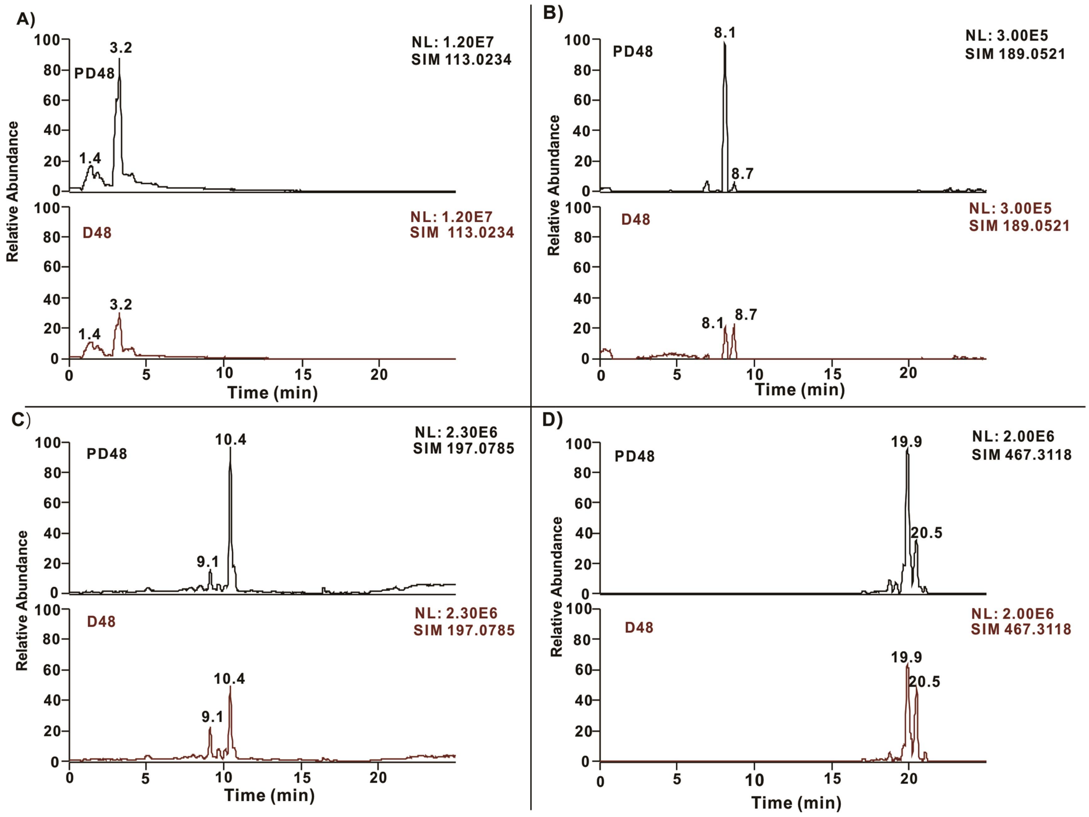

Figure S8. The LC-MS Profiles of difference of metabolites in PD48 vs D48 experiments. (A) The LC-MS Profiles of 2-furoic acid (**1**) in PD48 vs D48 experiments. (B). The LC-MS Profiles of 3,4-dimethoxybenzaldehyde (**4**) in PD48 vs D48 experiments. (C) The LC-MS Profiles of 3,4-dimethoxybenzyl formate (**6**) in PD48 vs D48 experiments. (D) The LC-MS Profiles of 3β,5α,9α-trihydroxy-ergosta-7,22-dien-6-one (**4**) in PD48 vs D48 experiments.

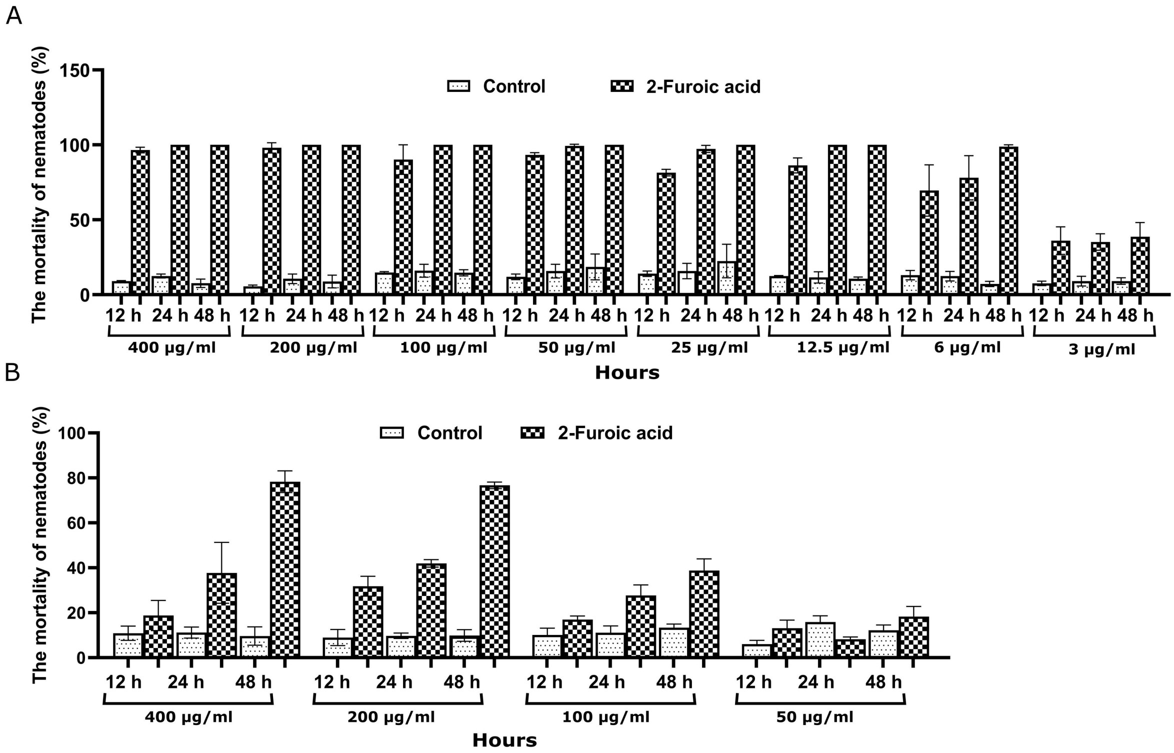

Figure S9. Nematicidal activity of 2-furoic acid. (A) Lethality of different concentrations of 2-furoic acid on *P. redivivus.* (B) Lethality of different concentrations of 2-furoic acid on *C. elegans*.

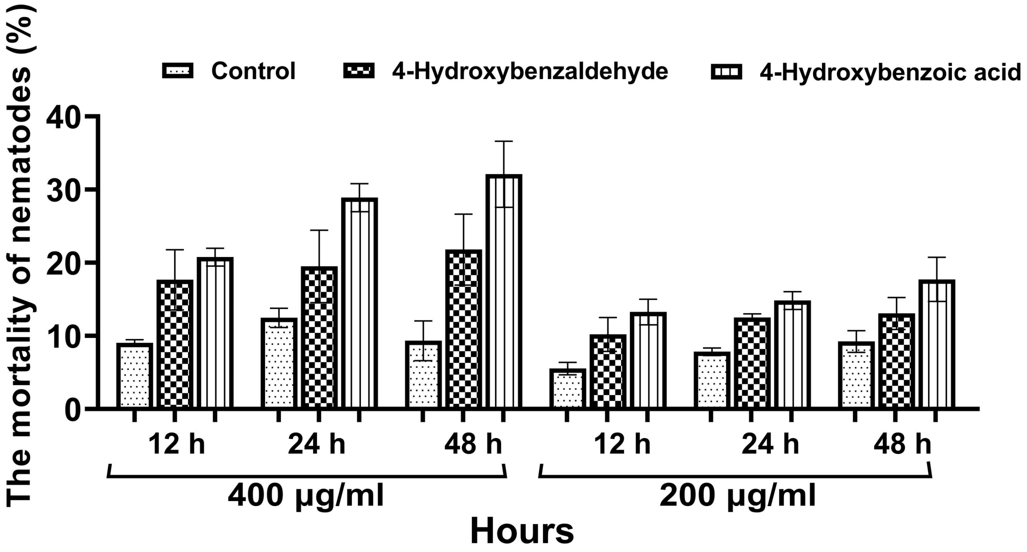

Figure S10. Nematicidal activity of 4-hydroxybenzoic acid (**2**) and 4-hydroxybenzaldehyde (**3**) on *P. redivivus*.

Table S1 Lengths of *D. haptotyla* YMF 1.03409 chromosomes

Group	Chromosome	Sequence Number	Sequence Length (bp)
Lachesis Group0	1	1	7783607
Lachesis Group1	2	1	4479033
Lachesis Group2	3	1	4364511
Lachesis Group3	4	1	3671591
Lachesis Group4	5	1	3741584
Lachesis Group5	6	1	3502361
Lachesis Group6	7	1	3587879
Lachesis Group7	8	1	3378366
Lachesis Group8	9	1	2389400
Lachesis Group9	10	1	2658696
Total Sequences Clustered (Ratio %)	-	10 (90.91)	39557028(99.37)
Total Sequences Ordered and Oriented (Ratio %)	-	10 (100)	39557028(100)

Table S2 Statistics of repeat elements in *D. haptotyla* YMF 1.03409 genome and MHA_v2

Type	YMF 1.03409			MHA_v2
	Number	Length	Rate (%)	Number
**Class I**	**327**	**254702**	**0.64**	**223**
Class I/DIRS	1	39	0	1
Class I/LINE	27	1715	0	3
Class I/LTR/Copia	69	94412	0.24	18
Class I/LTR/Gypsy	80	44081	0.11	64
Class I/PLE|LARD	64	101329	0.25	39
Class I/SINE	86	24124	0.06	98
**Class II**	**81**	**4490**	**0.01**	**91**
Class II/Helitron	9	647	0	2
Class II/TIR	58	2995	0.01	64
Class II/Unknown	14	1016	0	25

Table S6 Gene family contracted and expanded of *D. haptotyla* YMF 1.03409

ID	Description	P value	genes
**Expanded**			
ko00051	Fructose and mannose metabolism	0.02700623	Lachesis_group9G00083650.1
ko00270	Cysteine and methionine metabolism	0.04037071	Lachesis_group7G00082520.1
**Contracted**			
ko00900	Terpenoid backbone biosynthesis	5.27E-06	Lachesis_group1G00025140.1/ Lachesis_group4G00046920.1/ Lachesis_group7G00082450.1/ Lachesis_group7G00082990.1
ko02010	ABC transporters	1.14E-03	Lachesis_group0G00005700.1/ Lachesis_group9G00087740.1

Table S8 Number of gene cluster involved in biosynthesis of secondary metabolites in 12 species

Gene cluster no.	*D.hap*	*A.alt*	*A.fla*	*A.nid*	*B.bas*	*D.cio*	*D.bro*	*F.gra*	*M.ani*	*N.cra*	*O.oli*	*P.ory*
Indole	1	0	1	2	0	3	0	0	2	1	1	7
NRPS	5	10	1	9	8	3	0	8	11	6	1	14
Siderophore	1	0	1	0	0	3	0	1	1	0	1	0
T1PKS	8	3	3	18	3	6	4	3	14	6	4	14
T3PKS	1	1	1	0	0	1	1	1	0	1	1	2
Terpene	5	7	2	8	7	8	3	11	6	3	6	7
T1PKS-NRPS	1	3	0	2	3	1	1	2	8	0	0	5
T3PKS-T1PKS	0	0	0	0	0	0	0	0	0	0	0	0
Terpene-NRPS	0	0	0	0	0	0	0	1	0	0	0	0
Indole-NRPS	0	0	0	1	0	0	0	0	2	0	0	1
Indole-T1PKS	0	0	0	1	0	0	0	0	0	0	0	0
T1PKS-Terpene	0	0	0	1	0	0	0	0	1	0	0	0
Indole-Terpene	0	0	0	0	0	0	0	2	1	0	0	0
Other	1	1	1	3	0	0	0	0	4	1	1	1
Total number	23	25	10	45	21	24	9	29	50	18	15	51
Genome sizes	39.55	34.2	36.6	29.8	37.1	43.1	35.4	38	38.5	41.1	40	40.9

Table S13 Twenty-eight known compounds confidently annotated in PD48 vs D48 experiments

Name	m/z	Formula	Ion mode	Calc. MW	Ratio PD24/D24	Ratio PD48/D48
2-Furoic acid	113.0234	$C_5H_4O_3$	$[M+H]^{+1}$	113.0233	1.064	750.473
6-Methylsalicylic acid	153.0543	$C_8H_8O_3$	$[M+H]^{+1}$	153.0546	1.064	27.13
Phomalactone	155.0676	$C_8H_{12}O_4$	$[M+H]^{+1}$	155.0702	1.064	47.208
Gibepyrone A	165.0907	$C_{10}H_{13}O_2$	$[M+H]^{+1}$	165.0910	0.894	6.257
Terreic acid	177.0160	$C_7H_6O_4$	$[M+Na]^{+1}$	177.0158	1.064	15.005
(-)-Mellein	179.0699	$C_{10}H_{10}O_3$	$[M+H]^{+1}$	179.0703	1.064	64.022
Acetyl Sumiki's acid	185.0438	$C_8H_8O_5$	$[M+H]^{+1}$	185.0445	1.064	80.79
3,4-Dimethoxybenzaldehyde	189.0521	$C_9H_{10}O_3$	$[M+Na]^{+1}$	189.0521	7.244	88.808
6-Hydroxymellein	195.0636	$C_{10}H_{10}O_4$	$[M+H]^{+1}$	195.0652	1.064	44.453
3,4-dimethoxybenzyl formate	197.0785	$C_{10}H_{12}O_4$	$[M+H]^{+1}$	197.0808	1.064	54.935
Leucyl-Proline	229.1543	$C_{11}H_{20}N_2O_3$	$[M+H]^{+1}$	229.1547	1.064	3.835
Isoflavipucine	238.1062	$C_{12}H_{15}NO_4$	$[M+H]^{+1}$	238.1060	1.064	33.409
6-*O*-Methylpapyracon B	283.1511	$C_{15}H_{22}O_5$	$[M+H]^{+1}$	283.1540	1.096	493.761
Paganin B	299.1458	$C_{15}H_{22}O_6$	$[M+H]^{+1}$	299.1480	1.411	867.778
Phomoarcherin C	371.2209	$C_{23}H_{30}O_4$	$[M+H]^{+1}$	371.2217	1.06	36.608
9α-Methoxycortisol	393.2265	$C_{22}H_{32}O_6$	$[M+H]^{+1}$	393.2272	1.028	27.093
Aspernidgulene B2	403.2466	$C_{24}H_{34}O_5$	$[M+H]^{+1}$	403.2479	1.07	25.395
Calvasterol A	423.2886	$C_{28}H_{38}O_3$	$[M+H]^{+1}$	423.2894	1.426	20.193
Norselic acid C	425.3039	$C_{28}H_{40}O_3$	$[M+H]^{+1}$	425.3050	1.426	157.406
Stoloniferone A	427.3197	$C_{28}H_{42}O_3$	$[M+H]^{+1}$	427.3207	0.901	108. 036
Alternapyrone	429.3352	$C_{28}H_{44}O_3$	$[M+H]^{+1}$	429.3363	1.004	67.878
Farnesylemefuranone C	443.2042	$C_{23}H_{32}O_7$	$[M+Na]^{+1}$	443.2040	0.885	37.823
3β,5α-dihydroxy-14β,15β-epoxyergosta-7,22-diene-6-one	443.3146	$C_{28}H_{42}O_4$	$[M+H]^{+1}$	443.3156	1.175	71.458
3β,5α,9α-trihydroxy-ergosta-7,22-dien-6-one	467.3118	$C_{28}H_{44}O_4$	$[M+Na]^{+1}$	467.3132	1.093	33.93
Norselic acid E	495.3099	$C_{31}H_{42}O_5$	$[M+H]^{+1}$	495.3105	0.914	10.057
1-Oleoylglycerophosphocholine	522.3542	$C_{26}H_{52}NO_7P$	$[M+H]^{+1}$	522.3554	14.094	64.357
Lucentamycin C	523.3602	$C_{26}H_{46}N_6O_5$	$[M+H]^{+1}$	523.3602	0.994	105.569
Eschscholtzxanthone	565.4063	$C_{40}H_{52}O_2$	$[M+H]^{+1}$	565.4040	1.076	31.439

Methods S1: **Mycelium sample prepared for genome sequencing**

Preparation of spore suspension: *D. haptotyla* YMF1.03409 was inoculated on PDA medium at 28 °C for 20 days. Subsequently, the spores were washed off the surface of the mycelium with sterile water on an ultra-clean table to obtain a spore suspension. 2) Acquisition of vegetative hyphae: the spore solution was added to potato dextrose broth (PDB) medium and incubated at 28 °C for 14 days at 180 rpm to obtain vegetative hyphae. The vegetative hyphae were separated from the fermentation broth by filtration, and the mycelium was rinsed thrice with double distilled H_2O. Subsequently, the vegetative hyphae were drained as much as possible with filter paper sheets, and finally, the mycelium was snap-frozen with liquid nitrogen and stored at -80 °C in a refrigerator.

Methods S2: **Genome sequencing and assembly**

DNA extraction was performed using the CTAB binding QIAGEN Genomic-tip 20/G and its quality was estimated by Qubit system. 7.23 Gbp raw data were obtained by Nanopore long-read sequencing platform (Oxford Nanopore Technologies, UK). An in-house script was used to trim the raw sequencing data and 6.98 Gbp clean data were retained. Besides, 4.49 Gbp short-read data was obtained by Illumina HiSeq 4000 system (Illumina, USA).

Hi-C libraries were created from ~2 million *D. haptotyla* YMF 1.03409 cells. These cells were cross-linked and digested by HindIII, the restriction enzyme. The DNA fragment sticky ends were then biotinylated and ligated into chimeric circles. After enrichment, the chimeric circle was sheared and processed into sequencing libraries. The libraries were sequenced by Illumina HiSeq X Ten (Illumina). After quality-check and trim procedures, 7.41 Gbp clean Hi-C data was retained.

Canu v1.5 was used to assembly the clean Nanopore reads into primary contigs. Pilon was used to polish the contigs by NGS short reads. Hi-C data were then mapped to the polished contigs by bwa. LACHESIS was used to generate the final *D. haptotyla* YMF 1.03409 assembly. To assess the identity between our new assembly and previous published scaffold-level MHA_v2 assembly, we used NUCMER to perform whole-genome alignment. The identity dot figure was painted by mummerplot function in MUMmer.

Methods S3: **Gene and genomic components prediction**

The gene prediction was performed by MAKER-2.31.10. Their basic functional annotations were obtained by BLAST and Blast2Go. Besides, we also used BLAST to annotate genes on TCDB and PHI databases. CAZy annotation were obtained by hmmer.

We built a repetitive sequence database of the genome assembly based on the principles of structural prediction and de novo prediction, which was implemented by LTR_FINDER v1.05, MITE-Hunter, RepeatScout v1.0.5, and PILER-DF v2.4. The database was then classified with PASTEClassifier, and then cooperated with Repbase to generate the final repetitive database. Then the RepeatMasker v4.0.6 software is used to make repeat predictions based on the built repeating repetitive sequence database.

Methods S4: **Comparative genomics analysis**

Gene family clustering was first performed based on all amino acid sequences of the selected species using orthofinder v2.3.12 software (Emms & Kelly, 2019), where MUSCLE v3.8.1551 software was used to do align protein sequences. Using perl scripts to count and plot Venn diagrams. GO (Ashburner *et al.*, 2000) and KEGG (Kanehisa, 2000) analysis were performed using the R package clusterProfiler (Wu *et al.*, 2021) based on shared gene and functional annotations. Multiple sequence alignment was performed for each single-copy gene family using the software muscle (version: 3.8.31) (Edgar, 2004), followed by trimal (version: v1.4. rev 22; parameter: -gt 0.2) (Capella-Gutierrez *et al.*, 2009). Finally, a phylogenetic tree of ML species based on supergene was constructed using RAxML (version: 8.2.10) (Stamatakis, 2014) software (model: PROTGAMMAWAG). Based on the topology of the phylogenetic tree and the table of fossil time nodes (as above), the mcmctree subroutine (version: 4.9; parameters: nsample=3000000; burnin=8000000; seqtype=0; model=4) of the PAML (Yang, 2007) package was used to estimate the divergence of the selected species time. The CAFE5 (Han *et al.*, 2013, p. 3) software was used to estimate the number of gene family members for each branch's ancestor using a birth-mortality model based on species evolutionary trees and gene family clustering results to predict the contraction and expansion of the species' gene family relative to the ancestor.

Methods S5: **RNA-seq analysis**

After passing the assay, total RNA samples were enriched with magnetic beads with Oligo (dT) for eukaryotic mRNA, fragmentation buffer was added to fragment the mRNA, and the

fragmented mRNA was used as a template to synthesize the first strand of cDNA with six-base random primers, then added buffer, dNTPs (A, U, G, C), RNase H and DNA polymerase I to synthesize cDNA second strand. The double-stranded cDNA was then purified by magnetic beads and eluted with EB buffer, and the eluted double-stranded cDNA was processed for end repair, base A addition and sequencing junction addition. The entire library preparation was then completed by using magnetic beads for fragment size selection, degradation of U-containing chains, and PCR amplification.

To investigate the expression landscape more comprehensively, we re-predicted the gene with an ensemble method. Genscan, Augustus v2.4, GlimmerHMM v3.0.4, GeneID v1.4, and SNAP were used to perform ab initio gene prediction. GeMoMa was used to perform homologous protein-based gene prediction. The results were integrated by EVM v1.1.1. 11,073 genes were finally identified.

RNA-seq libraries were sequenced by Illumina HiSeq systems. The reads length was pair-end 150 bp. Transcriptome quantification was performed by Salmon with default parameters. The R package DESeq2 was used for differential expressed genes (DEGs) analysis. The DEGs cuf-off was set as p-value < 0.05 and absolute log (Fold Change) > 0.5. R package mfuzz was used for time-series gene expression trend analysis.

Methods S6: **Metabolomic data acquisition and statistical analysis**

Untargeted LC-MS metabolomics was performed on a Dionex UltiMate 3000 LC system coupled with a Q-Exactive Orbitrap mass spectrometer (San Jose, CA) (Thermo, Bremen, Germany). All samples were separated on a XTerra® MS C18 (150 mm × 3.9 mm, Waters) with a particle size of 5 µm at an LC flow rate of 1.0 ml/min with a flow split ratio of 0.3 to the ionization source, and a column temperature of 40°C. Mobile phase A was 0.1% formic acid in water, and mobile phase B was 0.1% formic acid in methanol. The 30 min gradient for positive ESI mode was set as follows: 0–5 min, 2% solvent B; 5–23 min, 2–99% solvent B; 23–28 min, 99% solvent B; and 28.1–35 min, 2% solvent B. The injection volume was 10 µl, and each sample was injected in triplicates. The LC–MS instrument was controlled using Thermo Scientific Xcalibur 4.4 software.

Compound Discoverer (CD version 3.3, Thermo Fisher Scientific) software was used for metabolomics data analysis of raw data file. Three blank samples were used for background

subtraction and noise removal during the pre-processing step. The data were analyzed in six groups (P24, P48, D24, D48, PD24, and PD48). The QC samples were used to normalize each individual compound and compensate for instrumental drift. For analysis of the data on metabolite variation in the six groups, simple univariate statistical analyses were carried out on log_2-transformed data using a paired t-test. Volcano plots were created using these data, with a threshold of $p < 0.01$ and absolute log_2 fold-change of > 1 set for defining a notable change in compound abundance between PD48 and D48 group. All components were searched and compounds are annotated following the references (Hu *et al.*, 2022; Qu *et al.*, 2023).

Methods S7: **Purification and characterization of 2-furoic acid from *D. haptotyla* YMF1.03409**

Thin-layer chromatography (TLC) was performed using precoated silica gel GF254 plates (Qingdao Marine Chemical Inc., China) with various solvent systems. Column chromatography was performed using silica gel (Qingdao Marine Chemical Inc., China) and Sephadex LH-20 (Amersham Biosciences, Uppsala, Sweden). The 31 d culture products of *D. haptotyla* YMF1.03409 cultured on 40 L Rice medium (60 g of rice, 0.3 g of $(NH_4)_2SO_4$ and pork liver, 30 ml of H_2O) was extracted six times with organic reagent mixture (ethyl acetate: methanol: acetic acid = 80:15:5, *v*/*v*/*v*). The filtrate was evaporated on a rotary evaporator under reduced pressure to obtain the crude extract (35.64 g). Then crude extract was further gradient elution on silica gel column (200−300 mesh) with petroleum ether/ethyl acetate (*v*/*v*), trichloromethane/methanol (*v*/*v*) to yield 12 fractions (Fr.1-12). Fr.8 (3.167 g) was further separated on trichloromethane/methanol (1:1, *v*/*v*) gel column to yield 13 fractions (Fr.8.1-13). Fr.8.8 (147 mg) was subjected to silica gel column with petroleum ether/ethyl acetate (20:1, *v*/*v*) to yield **1** (7 mg). Fr.8.5 (237 mg) was separated on a silica gel column (200-300 mesh) using trichloromethane/acetone (100:1→8:2, *v*/*v*) to give 13 fractions (Fr.8.5.1-13). Fr.8.5.2 (17 mg) was purified on a Sephadex LH-20 (methanol) to obtain **5** (4 mg). Fr.7 (865 mg) was subjected to a column of silica gel (200-300 mesh) using trichloromethane/methanol (200:1→10:1, *v*/*v*) to give 14 fractions (Fr.7.1-14). Fr.7.8 (20 mg) was purified on a column of silica gel (200-300 mesh) eluting by petroleum ether/acetone (50:1→8:2, *v*/*v*) to give 8 fractions (Fr.7.8.1-8). Fr.7.8.6 (7 mg) was further loaded on a Sephadex LH-20 (acetone) to obtain **2** (2 mg). Fr.5 (589 mg) was separated on a silica gel column (200-300 mesh) using trichloromethane/acetone (100:1→8:2, *v*/*v*) to give 17 fractions (Fr.5.1-17). Fr.5.5 (10 mg) was separated on a Sephadex

LH-20 eluting with acetone and further separated on a silica gel column to yield **3** (1mg). Fr.7.4 (5 mg) was further separated on acetone gel column to yield **4** (1 mg). Fr.7.5 (87 mg) was loaded on a silica gel column (200-300 mesh) and then purified on a Sephadex LH-20 (acetone) to produce **6** (1 mg). Fr.10 (3 g) was separated on a silica gel column (200-300 mesh) with trichloromethane/methanol (1:1, *v*/*v*) gel column to yield 5 fractions (Fr.10.1-5). Fr.10.6 (50 mg) was separated on a Sephadex LH-20 (methanol) to yield **7** (3 mg). Fr.8.6 (47 mg) was loaded on a silica gel column (200-300 mesh) and then purified on a Sephadex LH-20 (acetone) to produce **8** (2 mg). Fr.3 (2.085 g) was chromatographed over a silica gel column (200-300 mesh) and eluted with petroleum ether/ethyl acetate (20:1→7:3, *v*/*v*) to produce 11 fractions (Fr.3.1-11). Fr.3.2 (408 mg) was chromatographed over a silica gel column (200-300 mesh) and eluted with petroleum ether/ethyl acetate (100:1→6:4, *v*/*v*) to produce 8 fractions (Fr.3.2.1-8). Fr.3.2.7 (15 mg) was subjected on a silica gel column (200-300 mesh) with petroleum ether/acetone (20:1, *v*/*v*) to yield **9** (2 mg). Fr.12 (1.872 g) was separated on a Sephadex LH-20 (methanol) eluting with methanol to give 5 fractions (Fr.12.1-5). Fr.12.5 (160 mg) was loaded on a silica gel column (200-300 mesh) with trichloromethane/methanol (100:1→8:2, *v*/*v*) and then purified on a Sephadex LH-20 (methanol) to produce **10** (1 mg). Fr.8.6.2 (5 mg) was purified on a Sephadex LH-20 (acetone) to produce **11** (2 mg). Fr.4 (188 mg) was chromatographed over a silica gel column (200-300 mesh) and eluted with petroleum ether/ethyl acetate (50:1→6:4, *v*/*v*) to produce 7 fractions (Fr.4.1-7). Fr.4.3 (21 mg) was purified on a column of silica gel (200-300 mesh) and eluting by petroleum ether/acetone (200:1→10:1, *v*/*v*) to obtain 3 fractions (Fr.4.3.1 - 3). Fr.3.2.4 (207 mg) was subjected on a silica gel column (200-300 mesh) with petroleum ether/ethyl acetate (20:1, *v*/*v*) to yield **12** (2 mg). Fr.5 (589 mg) was separated on a silica gel column (200-300 mesh) using trichloromethane/acetone (100:1→8:2, *v*/*v*) to give 17 fractions (Fr.5.1-17). Fr.5.17 (8 mg) was separated on a Sephadex LH-20 (acetone) to yield **13** (1 mg). Fr.9 (428 mg) was further separated on trichloromethane/methanol (1:1, *v*/*v*) gel column to yield 7 fractions (Fr.9.1-7). Fr.9.3 (74 mg) was separated on a silica gel column (200-300 mesh) with trichloromethane/methanol (50:1→20:1, *v*/*v*) to give 9 fractions (Fr.9.3.1-9). Fr.9.3.7 (2 mg) was purified on a Sephadex LH-20 (methanol) to obtain **14** (1 mg).

Data S1: Spectroscopic data of obtained metabolites

2-Furoic acid (**1**)**,** white solid; ESI-MS: 113 $[M + H]^+$; ^{1}H-NMR (CD_3OD, 600 MHz) δ_H: 6.58 (1H, t, J = 1.6 Hz), 7.19 (1H, brd, J = 3.5 Hz), 7.71 (1H, brs); ^{13}C-NMR (CD_3OD, 150 MHz) δ_C: 112.9 (d), 119.0 (d), 146.4 (s), 148.0 (d), 161.8 (s) (Cui *et al.*, 2002).

4-Hydroxybenzoic acid (**2**)**,** white solid; ESI-MS: 139 $[M + H]^+$; ^{1}H-NMR (CD_3OD, 600 MHz) δ_H: 6.81 (1H, d, J = 8.8 Hz), 7.87 (1H, d, J = 8.2 Hz); ^{13}C-NMR (CD_3OD, 150 MHz) δ_C: 116.0 (d), 122.8 (s), 133.0 (d), 163.4 (s), 170.2 (d).

4-Hydroxybenzaldehyde (**3**)**,** colorless amorphous; ESI-MS: 121 $[M - H]^-$; ^{1}H-NMR (CD_3COCD_3, 600 MHz) δ_H: 7.00 (2H, d, J = 8.3 Hz), 7.80 (2H, d, J = 8.5 Hz), 9.37 (1H, s), 9.85 (1H, s); ^{13}C-NMR (CD_3COCD_3, 150 MHz) δ_C: 116.6 (d), 132.8 (d), 129.5 (s), 164.3 (s), 190.9 (d) (Kim *et al.*, 2003).

3,4-Dimethoxy-benzaldehyde (**4**)**,** colorless amorphous; ESI-MS: 189 $[M + Na]^+$; ^{1}H-NMR ($CDCl_3$, 600 MHz) δ_H: 3.47 (1H, s), 3.94 (3H, s), 3.97 (3H, s), 6.99 (1H, d, J = 8.2 Hz), 7.41 (1H, s), 7.47 (1H, d, J = 8.9 Hz); ^{13}C-NMR ($CDCl_3$, 150 MHz) δ_C: 56.0 (q), 56.2 (q), 108.9 (d), 110.3 (d), 126.9 (d), 130.1 (s), 149.6 (s), 154.9 (s), 190.9 (s) (Maddani & Prabhu, 2008).

3,4-Dimethoxy-benzenemethanol (**5**), white solid; ESI-MS: 191 $[M + Na]^+$; ^{1}H-NMR ($CDCl_3$, 600 MHz) δ_H: 3.88 (3H, s), 3.89 (3H, s), 4.63 (2H, d, J = 5.8 Hz), 6.85 (1H, d, J = 8.1 Hz), 6.90 (1H, d, J = 8.1 Hz), 6.93 (1H, s); ^{13}C-NMR ($CDCl_3$, 150 MHz) δ_C: 55.8 (q), 55.9 (q), 65.3 (t), 110.4 (d), 111.0 (d), 119.4 (d), 133.5 (s), 148.6 (s), 149.1 (s) (O'Byrne *et al.*, 2010).

3,4-Dimethoxybenzyl formate (**6**), yellow solid; ESI-MS: 216 $[M + Na]^+$; ^{1}H-NMR ($CDCl_3$, 600 MHz) δ_H: 3.89 (3H, s), 3.90 (3H, s), 5.14 (2H, s), 6.86 (1H, d, J = 8.2 Hz), 6.90 (1H, d, J = 1.6 Hz), 6.95 (1H, dd, J = 1.6, 8.2), 8.13 (1H, s); ^{13}C-NMR ($CDCl_3$, 150 MHz) δ_C: 55.88 (q), 55.92 (q), 65.8 (t), 111.0 (d), 111.8 (d), 121.4 (d), 127.6 (s), 149.0 (s), 149.3 (s), 160.9 (d) (Zeng *et al.*, 2009).

4-Hydroxyisophthalic acid (**7**)**,** Orange-red solid; ESI-MS: 205 $[M + Na]^+$; ^{1}H-NMR (CD_3OD, 600 MHz) δ_H: 8.56 (1H, s), 8.08 (1H, d, J = 8.8 Hz), 6.98 (1H, d, J = 8.3 Hz); ^{13}C-NMR (CD_3OD, 150 MHz) δ_C: 115.5 (s), 118.3 (d), 122.7 (s), 134.2 (d), 137.3 (d), 166.9 (s), 169.0 (s), 173.4 (s) (Wittmann *et al.*, 2010).

Trametol (**8**)**,** white solid; ESI-MS: 239 $[M + Na]^+$; 241 $[M + Na]^+$; ^{1}H-NMR (CD_3OD, 600 MHz) δ_H: 1.09 (1H, d, J = 6.4 Hz), 3.90 (3H, s), 4.00 (1H, m), 4.61 (1H, m), 6.93 (1H, J = 8.5 Hz), 7.23 (1H, J = 8.5 Hz), 7.40 (1H, s); ^{13}C-NMR (CD_3OD, 150 MHz) δ_C: 17.4 (q), 56.2 (q),

71.1 (d), 76.5 (d), 111.8 (d), 122.8 (s), 126.4 (d), 128.6 (d), 133.5 (s), 154.6 (s) (Brambilla *et al.*, 1995).

1,10-Dimethoxybenzo[c]cinnoline (**9**), colorless amorphous; ESI-MS: 263 $[M + Na]^+$; ^{1}H-NMR ($CDCl_3$, 600 MHz) δ_H: 4.18 (6H, s), 7.10 (2H, d, J = 8.0 Hz), 7.75 (2H, t, J = 8.0 Hz), 8.01 (2H, d, J = 8.0 Hz); ^{13}C-NMR ($CDCl_3$, 150 MHz) δ_C: 56.5 (q), 106.9 (d), 122.0 (d), 130.2 (d), 136.9 (s), 143.0 (s), 154.9 (s) (Wang *et al.*, 2007).

1-(2'-Deoxy-*β*-D-erythro-pentofuranosyl)-1*H*-1,2,4-triazole (**10**), white solid; ESI-MS: 186 $[M + H]^+$; ^{1}H-NMR (CD_3OD, 600 MHz) δ_H: 8.32 (1H, s), 8.17 (1H, s), 6.43 (1H, t, J = 6.2 Hz), 4.58 (1H, m), 4.06 (1H, m), 3.85 (1H, dd, J = 12.4, 2.9 Hz), 3.75 (1H, dd, J = 12.3, 3.3Hz), 2.82 (1H, m), 2.42 (1H, m); ^{13}C-NMR (CD_3OD, 150 MHz) δ_C: 41.6 (t), 63.7 (t), 73.1 (d), 87.2 (d), 89.9 (d), 141.6 (d), 153.5 (d) (Huang *et al.*, 2011).

Methyl-3,4-dihydroxybutyrate (**11**), colorless amorphous; ESI-MS: 133 $[M - H]^-$; ^{1}H-NMR (CD_3OD, 600 MHz) δ_H: 2.36 (1H, d, J = 17.7 Hz), 2.82 (1H, dd, J = 5.9, 17.7 Hz), 3.51 (2H, m), 3.67 (3H, s), 4.03 (1H, m); ^{13}C-NMR (CD_3OD, 150 MHz) δ_C: 39.6 (t), 52.1 (q), 66.6 (t), 70.1 (d), 173.9 (s) (White *et al.*, 2008).

β-Sitostenone (**12**), colorless amorphous; ESI-MS: 435 $[M + Na]^+$; ^{1}H-NMR ($CDCl_3$, 600 MHz) δ_H: 5.72 (1H, s, H-4), 1.18 (3H, s), 0.80-1.10 (12H, m), 0.71 (3H, s, H-18); ^{13}C-NMR ($CDCl_3$, 150 MHz) δ_C: 11.9 (q), 12.9 (q), 17.4 (q), 18.7 (q), 19.0 (q), 19.8 (q), 21.0 (q), 23.0 (t), 24.1 (t), 26.0 (t), 28.2 (t), 29.1 (t), 32.0 (t), 32.9 (t), 33.9 (t), 34.0 (t), 35.6 (t), 35.7 (t), 36.1 (t), 38.6 (t), 39.6 (s), 42.4 (s), 45.8 (d), 53.8 (d), 55.9 (d), 56.0 (d), 123.7 (d), 171.7 (s), 199.7 (s) (Prachayasittikul *et al.*, 2009).

(22*E*,24*R*)-Ergosta-5,7,22-trien-3β-ol (**13**), yellow solid; ESI-MS *m/z:* 397 $[M + H]^+$; ^{1}H-NMR (CD_3COCD_3, 600 MHz) δ: 3.66 (1H, m, H-3), 5.52 (1H, m, H-6), 5.36 (1H, m, H-7), 0.66 (3H, s), 0.93 (3H, s), 1.06 (3H, d, J = 6.7 Hz), 5.19 (1H, m, H-22), 5.25 (1H, *m*, H-23), 0.84 (3H, d, J = 6.5 Hz), 0.82 (3H, d, J = 6.4 Hz), 0.96 (3H, d, J = 6.9 Hz); ^{13}C-NMR (CD_3COCD_3, 150 MHz) δ: 39.2 (t), 32.8 (t), 70.2 (d), 41.3 (t), 141.3 (s), 119.9 (d), 117.4 (d), 141.6 (s), 47.1 (d), 38.0 (s), 21.7 (t), 39.9 (t), 43.0 (s), 55.2 (d), 23.7 (t), 29.5 (t), 56.5 (d), 12.3 (q), 16.6 (q), 39.9 (d), 21.5 (q), 136.6 (d), 132.7 (d), 43.7 (d), 33.8 (d), 20.3 (q), 19.9 (q), 18.1 (q) (Smith, 1977).

3β,5α,9α-Trihydroxy-ergosta-7,22-dien-6-one (**14**), colorless amorphous; ESI-MS *m/z*: 445 $[M + H]^+$; ^{1}H-NMR ($CDCl_3$, 600 MHz) δ: 5.63 (1H, d, J = 1.8 Hz, H-7), 0.90 (3H, d, J = 6.7 Hz, H-21), 1.25 (3H, d, J = 5.9 Hz, H-21), 1.25 (3H, s, H-19), 0.85 (3H, d, J = 6.6 Hz, H-27), 0.82

(3H, d, J = 6.6 Hz, H-26), 0.80 (3H, d, J = 6.2 Hz, H-28); ^{13}C-NMR ($CDCl_3$, 150 MHz) δ: 26.6 (t), 31.0 (t), 67.8 (d), 37.1 (t), 80.2 (s), 200.1 (s), 120.9 (d), 165.0 (s), 76.2 (s), 42.0 (s), 29.1 (t), 36.1 (t), 46.2 (s), 52.8 (d), 23.4 (t), 26.6 (t), 57.4 (d), 12.6 (q), 20.1 (q), 34.4 (d), 20.1 (q), 136.7 (d), 133.6 (d), 41.7 (d), 42.7 (d), 18.2 (q), 21.6 (q), 20.5 (q) (Cai *et al.*, 2013).

References

Ashburner M, Ball CA, Blake JA, Botstein D, Butler H, Cherry JM, Davis AP, Dolinski K, Dwight SS, Eppig JT, *et al.* 2000. Gene Ontology: tool for the unification of biology. *Nature Genetics* **25**: 25–29.

Brambilla U, Nasini G, de Pava OV. 1995. Secondary mold metabolites, Part 49. Isolation, structural elucidation, and biomimetic synthesis of trametol, a new 1-arylpropane-1,2-diol produced by the fungus *Trametes* sp. *Journal of Natural Products* **58**: 1251–1253.

Cai H, Liu X, Chen Z, Liao S, Zou Y. 2013. Isolation, purification and identification of nine chemical compounds from *Flammulina velutipes* fruiting bodies. *Food Chemistry* **141**: 2873–2879.

Capella-Gutierrez S, Silla-Martinez JM, Gabaldon T. 2009. trimAl: a tool for automated alignment trimming in large-scale phylogenetic analyses. *Bioinformatics* **25**: 1972–1973.

Cui Y jun, Liu P, Chen R yun. 2002. Studies on the chemical constituents of *Spatholobus suberectus* Dunn. *Yao Xue Xue Bao = Acta Pharmaceutica Sinica* **37**: 784–787.

Edgar RC. 2004. MUSCLE: multiple sequence alignment with high accuracy and high throughput. *Nucleic Acids Research* **32**: 1792–1797.

Emms DM, Kelly S. 2019. OrthoFinder: phylogenetic orthology inference for comparative genomics. *Genome Biology* **20**: 238.

Han MV, Thomas GWC, Lugo-Martinez J, Hahn MW. 2013. Estimating gene gain and loss rates in the presence of error in genome assembly and annotation using CAFE 3. *Molecular Biology and Evolution* **30**: 1987–1997.

Hu Q-Y, Pu X-J, Li G-H, Li C-Q, Lei H-M, Zhang K-Q, Zhao P-J. 2022. Identification and mechanism of action of the global secondary metabolism regulator SaraC in *Stereum hirsutum* (RP de Vries, Ed.). *Microbiology Spectrum* **10**: e02624-22.

Huang R, Zhou X, Peng Y, Yang X, Xu T, Liu Y. 2011. Nucleosides from the marine sponge *Callyspongia* sp. *Chemistry of Natural Compounds* **46**: 1010–1011.

Kanehisa M. 2000. KEGG: Kyoto encyclopedia of genes and genomes. *Nucleic Acids Research* **28**: 27–30.

Kim H, Ralph J, Lu F, Ralph SA, Boudet A-M, MacKay JJ, Sederoff RR, Ito T, Kawai S, Ohashi H, *et al.* 2003. NMR analysis of lignins in CAD-deficient plants. Part 1. Incorporation of hydroxycinnamaldehydes and hydroxybenzaldehydes into lignins. *Organic & Biomolecular Chemistry* **1**: 268–281.

Maddani M, Prabhu KR. **2008**. A chemoselective aerobic oxidation of benzylic azides catalyzed by molybdenum xanthate in an aqueous medium. *Tetrahedron Letters* **49**: 4526–4530.

O'Byrne A, Murray C, Keegan D, Palacio C, Evans P, Morgan BS. **2010**. The thio-adduct facilitated, enzymatic kinetic resolution of 4-hydroxycyclopentenone and 4-hydroxycyclohexenone. *Organic & Biomolecular Chemistry* **8**: 539–545.

Prachayasittikul S, Suphapong S, Worachartcheewan A, Lawung R, Ruchirawat S, Prachayasittikul V. **2009**. Bioactive metabolites from *Spilanthes acmella* Murr. *Molecules* **14**: 850–867.

Qu S-L, Xie J, Wang J-T, Li G-H, Pan X-R, Zhao P-J. **2023**. Activities and metabolomics of *Cordyceps gunnii* under different culture conditions. *Frontiers in Microbiology* **13**: 1076577.

Smith WB. **1977**. The carbon-13 spectra of steroids on the way to ecdysone. *Organic Magnetic Resonance* **9**: 644–648.

Stamatakis A. **2014**. RAxML version 8: a tool for phylogenetic analysis and post-analysis of large phylogenies. *Bioinformatics* **30**: 1312–1313.

Wang K, Guo L, Zou Y, Li Y, Wu J. **2007**. 4849F, a new metabolite produced by the *Streptomyces* sp. 4849 as an Inhibitor of IL-4 receptor. *The Journal of Antibiotics* **60**: 325–327.

White JD, Lincoln CM, Yang J, Martin WHC, Chan DB. **2008**. Total synthesis of solandelactones A, B, E, and F exploiting a tandem petasis−claisen lactonization strategy. *The Journal of Organic Chemistry* **73**: 4139–4150.

Wittmann S, Schätz A, Grass RN, Stark WJ, Reiser O. **2010**. A recyclable nanoparticle-supported palladium catalyst for the hydroxycarbonylation of aryl Halides in water. *Angewandte Chemie International Edition* **49**: 1867–1870.

Wu T, Hu E, Xu S, Chen M, Guo P, Dai Z, Feng T, Zhou L, Tang W, Zhan L, *et al.* 2021. clusterProfiler 4.0: A universal enrichment tool for interpreting omics data. *The Innovation* **2**: 100141.

Yang Z. **2007**. PAML 4: phylogenetic analysis by maximum likelihood. *Molecular Biology and Evolution* **24**: 1586–1591.

Zeng T, Song G, Li CJ. **2009**. Separation, recovery and reuse of N-heterocyclic carbene catalysts in transesterification reactions. *Chemical Communications*: 6249.
